# ACLY Safeguards Copper Homeostasis to Sustain Brown Adipose Tissue Thermogenesis and Restrain Diet-Induced Obesity

**DOI:** 10.34133/research.1272

**Published:** 2026-05-07

**Authors:** Xin Liu, Lin Jia, Luwen Li, Jingnan Huang, Mengyun Hou, Zhijie Li, Dahong Yao, Yunfan Yang, Jigang Wang, Lingyun Dai

**Affiliations:** ^1^ Department of Geriatrics, Guangdong Provincial Clinical Research Center for Geriatrics, Shenzhen Clinical Research Center for Geriatrics, The First Affiliated Hospital of Southern University of Science and Technology, Shenzhen 518020, China.; ^2^ Department of Pharmacology, School of Medicine and SUSTech Homeostatic Medicine Institute (SHMI), Southern University of Science and Technology, Shenzhen 518055, China.; ^3^ Faculty of Pharmaceutical Sciences, Shenzhen University of Advanced Technology, Shenzhen 518107, China.; ^4^ College of Pharmacy, Shenzhen Technology University, Shenzhen 518118, China.; ^5^ State Key Laboratory of Medicinal Chemical Biology, College of Life Sciences, Nankai University, Tianjin 300071, China.; ^6^ Institute of Molecular and Cell Biology, Agency for Science, Technology and Research (A*STAR), Singapore 138673, Singapore.

## Abstract

Brown adipose tissue (BAT) plays a crucial role in maintaining energy balance and regulating glucose and lipid metabolism. Preserving BAT activity has been considered a potential strategy to mitigate metabolic dysfunctions such as diet-induced obesity (DIO). Here, we identified a pronounced copper overload in the BAT of DIO mice. Normalization of copper levels through copper chelation or cold exposure effectively restored the expression of the thermogenic effector protein UCP1, prevented BAT whitening, and improved systemic metabolic parameters. Proteomic profiling revealed ATP-citrate lyase (ACLY)—a key enzyme in acetyl-CoA synthesis and de novo lipogenesis—as a critical mediator in BAT copper homeostasis. ACLY expression was down-regulated in BAT of DIO mice but increased in cold-challenged or copper-chelated BAT. Loss of ACLY promoted NRF2 acetylation and nuclear translocation, leading to the transcriptional repression of the copper chaperone ATOX1, which exacerbated copper accumulation in BAT, resulting in BAT whitening and dysfunction. Connectivity Map screening identified succinylsulfathiazole (SST) as an upstream activator of ACLY expression. Both in vitro and in vivo administration of SST restored ACLY expression, corrected copper dysregulation, and re-established BAT activity. Collectively, these findings uncover a mechanism linking ACLY to copper homeostasis in BAT and highlight SST as a promising repurposed candidate for correcting obesity and BAT dysfunction-associated metabolic disorders.

## Introduction

Brown adipose tissue (BAT) serves as the primary site of nonshivering thermogenesis. This adaptive physiological response helps maintain body temperature in cold environments [1], largely through the activity of mitochondrial uncoupling protein 1 (UCP1) [2]. Initially recognized for its contribution to energy expenditure and thermogenesis in rodents in the mid-20th century [3], BAT is increasingly appreciated as a crucial metabolic regulator in humans [4]. Large-scale clinical analysis has revealed that BAT “whitening” and reduced activity in obese individuals are strongly associated with hyperglycemia, visceral fat accumulation, and aging [5]. Studies indicate that activation of BAT through cold exposure enhances energy expenditure, aids in body fat control in individuals with low or undetectable BAT activity [6], and improves insulin sensitivity in diabetic patients [7]. Furthermore, individuals with active BAT demonstrate a remarkably lower risk of developing cardiometabolic diseases [8]. The positive impact of BAT is also linked to a healthier metabolic phenotype in subjects with severe obesity [9]. Therefore, maintaining and activating BAT activity has been suggested to be a promising strategy for addressing obesity and the associated metabolic disorders [10–12].

Copper, a vital trace element essential for numerous physiological processes, plays a crucial role in maintaining health. An imbalance in copper levels—either deficiency or overload—can be detrimental, impairing various endocrine and metabolic functions [13]. There is a strong positive correlation between serum copper levels and obesity in humans [14–16], and the link between copper homeostasis and lipid metabolism has been reported [17]. Copper is indispensable for maintaining metabolic fuel in adipocytes [18]. Specifically, insufficient copper supply inactivates semicarbazide-sensitive amine oxidase (SSAO), a cuproprotein, triggering metabolic changes that result in adipocyte hypertrophy and triglyceride (TG) accumulation [18]. Evidence further suggests that copper overload in adipose tissue leads to increased lipolysis and DNA damage, resulting in accelerated aging and systemic metabolic disorders [19]. However, the importance of copper metabolism and BAT thermogenic function remains largely unexplored. Wang et al. [20] observed the dynamic changes of copper levels in cold-simulated BAT but not in subcutaneous white adipose tissue (sWAT), suggesting the involvement of copper homeostasis in BAT function; however, the molecular basis of this observation remains elusive.

Here, we discovered that the BAT of diet-induced obesity (DIO) mice displayed copper overload, accompanied by impaired thermogenic activity. Both cold exposure and the administration of copper chelator successfully re-established copper homeostasis within the BAT of DIO mice, alongside increased UCP1 expression and improved metabolic parameters. Guided by this discovery, we explored the mechanism linking copper imbalance to BAT dysfunction and identified ATP-citrate lyase (ACLY) as a central regulator of copper homeostasis in BAT. ACLY, primarily converting citrate, a product of the tricarboxylic acid (TCA) cycle, into acetyl-CoA, influences fatty acid synthesis through its expression or enzymatic activity [21]. Our findings uncovered a previously unrecognized role of ACLY in BAT biology. In addition, we explored the therapeutic potential of ACLY and discovered a repurposed pharmacological activator of ACLY, succinylsulfathiazole (SST), as a promising intervention to restore BAT thermogenic function and ameliorate obesity and systemic metabolic dysfunction.

## Results

### Copper overload contributes to BAT dysfunction and obesity in DIO mice

**Fig. 1.**
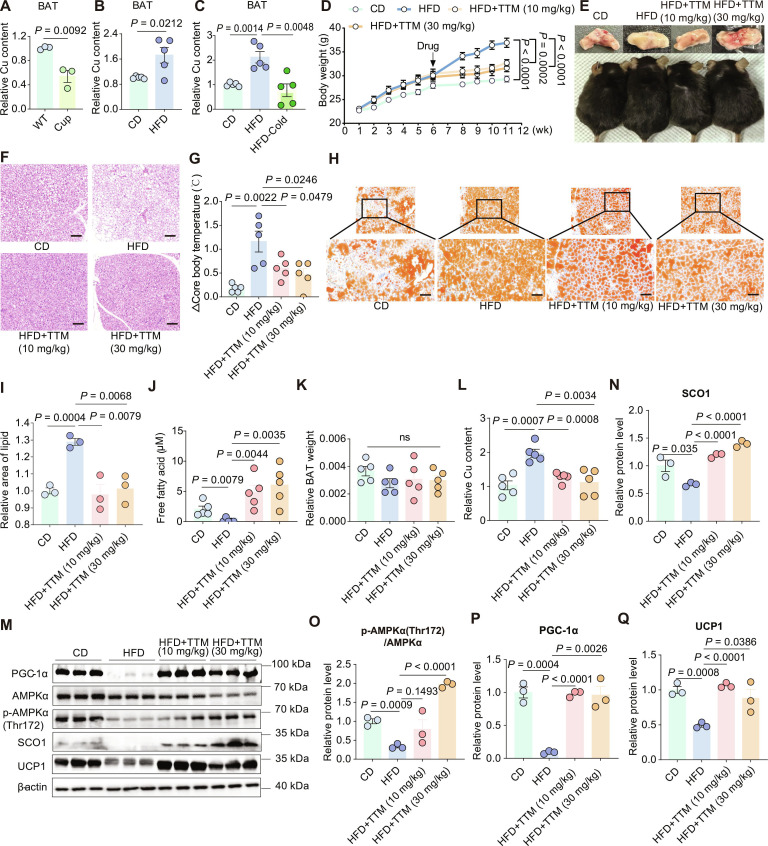
Copper overload contributes to brown adipose tissue (BAT) dysfunction and obesity in diet-induced obesity (DIO) mice. (A) Relative copper content in the BAT of wild-type (WT) mice and Cuprizone (Cup)-treated mice, with sample size, *n* = 3. (B) Relative copper content in the BAT of chow diet (CD) and DIO mice that were fed with high-fat diet (HFD), *n* = 5. (C) Relative copper content in the BAT of CD, DIO, and cold-exposed DIO mice, *n* = 5. (D) Changes in body weight across the CD, DIO, and tetrathiomolybdate (TTM)-treated groups, *n* = 6. Statistics for the overall effect represent the *P* values from a 2-way analysis of variance (ANOVA) followed by Dunnett’s test. (E) Visual representation of body shape and BAT morphology in each mouse group. (F) Hematoxylin–eosin (H&E) staining of BAT sections from each group of mice (scale bar: 20 μm). (G) The absolute value of the decrease in core body temperature (Δcore body temperature) in mice of each group after cold exposure, *n* = 5. (H and I) Oil Red O staining of BAT sections from mice in each group, along with quantification (scale bar: 40 μm). (J) Free fatty acid content in the BAT of mice from each group, *n* = 5. (K) The ratio of BAT mass to body weight in each group, *n* = 5. (L) Relative copper content in BAT of each group, *n* = 5. (M to Q) Western blot analysis and quantification of SCO1, p-AMPKα(Thr172), AMPKα, PGC-1α, and UCP1 protein expressions in the BAT of the mice across all groups, *n* = 3. Data are presented as the mean ± SEM of independent biological replicates; *P* < 0.05 represents a statistically significant effect, while ns represents no significant effect.

Our team initially observed that the adipose tissue in the interscapular region of mice subjected to a multiple sclerosis model, induced by the copper chelator Cuprizone, exhibited an appearance similar to that observed in high-fat diet (HFD)-fed mice, a model of DIO (Fig. S1A, top panel). It is essential to clarify that this appearance pertained primarily to the surrounding WAT rather than the BAT itself. Specifically, the BAT in the DIO mice exhibited signs of “whitening”, whereas the BAT within the Cuprizone-treated group resembled that of mice on a normal chow diet (CD) (Fig. S1A, bottom panel). Considering the role of Cuprizone as a copper chelator, we proceeded to measure the relative copper content in the BAT of both DIO mice and those treated with Cuprizone. Our analysis revealed a decrease in the relative copper content within the BAT of Cuprizone-treated mice (Fig. 1A), and, notably, for the first time, we discovered a copper overload within the BAT of DIO mice (Fig. 1B). Moreover, we observed increased relative copper content in the plasma and inguinal WAT (iWAT) of DIO mice (Fig. S1B and C), while no significant alterations were detected in the liver and epididymal WAT (eWAT) when compared to the CD group mice (Fig. S1D and E).

To explore the potential link between copper homeostasis in BAT and thermogenic activity, we exposed DIO mice to cold stimulation (4 °C, 1 h/d for 1 week). The intervention restored the relative copper content in the BAT of DIO mice to normal levels (Fig. 1C). These results suggest that copper overload may correlate with impaired BAT activity. To further validate this hypothesis, we administered a widely used copper chelator, tetrathiomolybdate (TTM), via oral gavage to DIO mice over 6 weeks (at dosages of 10 and 30 mg/kg, respectively). The TTM treatment significantly reduced both the rate and extent of weight gain in DIO mice (Fig. 1D), inhibited the whitening of BAT (Fig. 1E and F), alleviated the hypothermia induced by cold exposure (Fig. 1G), reduced lipid content in the BAT (Fig. 1H and I), and elevated levels of free fatty acid (FFA) within the BAT (Fig. 1J), despite no significant changes in the BAT-to-body weight ratio (Fig. 1K). The activation of BAT is associated with marked increases in FFA uptake and storage [22]; thus, these results suggested enhanced BAT activation after TTM treatment. Importantly, TTM treatment restored the relative copper content in the BAT of DIO mice to normal levels (Fig. 1L and Fig. S1F), demonstrating an essential role for copper homeostasis in BAT activation.

Furthermore, TTM administration did not adversely affect liver or kidney function (Fig. S1G to I); however, it did lead to reductions in fasting blood glucose (Fig. S1J), TG (Fig. S1K), and total cholesterol (T-CHO) levels (Fig. S1L) in DIO mice. Notably, a dose of 10 mg/kg of TTM effectively elevated the high-density lipoprotein cholesterol (HDL-C) levels (Fig. S1M). No significant impact was observed on low-density lipoprotein cholesterol (LDL-C) levels within the tested range (Fig. S1N). In addition, a dose of 10 mg/kg of TTM also effectively decreased creatine kinase MB isoenzyme (CK-MB) levels (Fig. S1O). Collectively, these findings suggest that TTM treatment alleviates copper overload, thereby counteracting obesity and ameliorating metabolic dysregulation in DIO mice.

### Copper overload inhibits the SCO1/AMPK/PGC-1α pathway

Prior research in the context of hepatocytes has identified synthesis of cytochrome C oxidase 1 (SCO1) as both a copper sensor and a copper-dependent signaling scaffold, in which copper level directly regulates the assembly of the SCO1/LKB1 (liver kinase B1)/AMPK (AMP-activated protein kinase) complex [23]. An adequate copper level is essential for AMPK activation, which decreases the acetylation of PGC-1α, promotes its translocation to the nucleus, and thereby initiates the transcription of downstream target genes [23]. Within BAT, PGC-1α serves as the primary regulator of the expression of UCP1, a key effector of thermogenesis [24]. To date, no studies have investigated the potential regulation of BAT function by copper ions through the SCO1/AMPK/PGC-1α pathway. In the present study, we examined the protein expression levels associated with this pathway in the BAT of the different mouse groups. Our findings indicated that the SCO1/AMPK/PGC-1α signaling pathway was notably suppressed in the BAT of the DIO mice (Fig. 1M). Strikingly, following TTM treatment, we observed a restoration in the expression levels of SCO1, p-AMPKα(Thr172)/AMPKα, and PGC-1α (Fig. 1N to P), alongside an increase in UCP1 expression (Fig. 1Q). Additionally, we treated the primary brown adipocytes with the combination of the copper ionophore Elesclomol (ES) and CuCl₂ to increase intracellular copper levels. Western blot analysis showed that the treatment of ES and CuCl₂ led to a decrease in the SCO1 protein expression (Fig. S1P).

### Down-regulation of ACLY expression causes copper overload in BAT

**Fig. 2.**
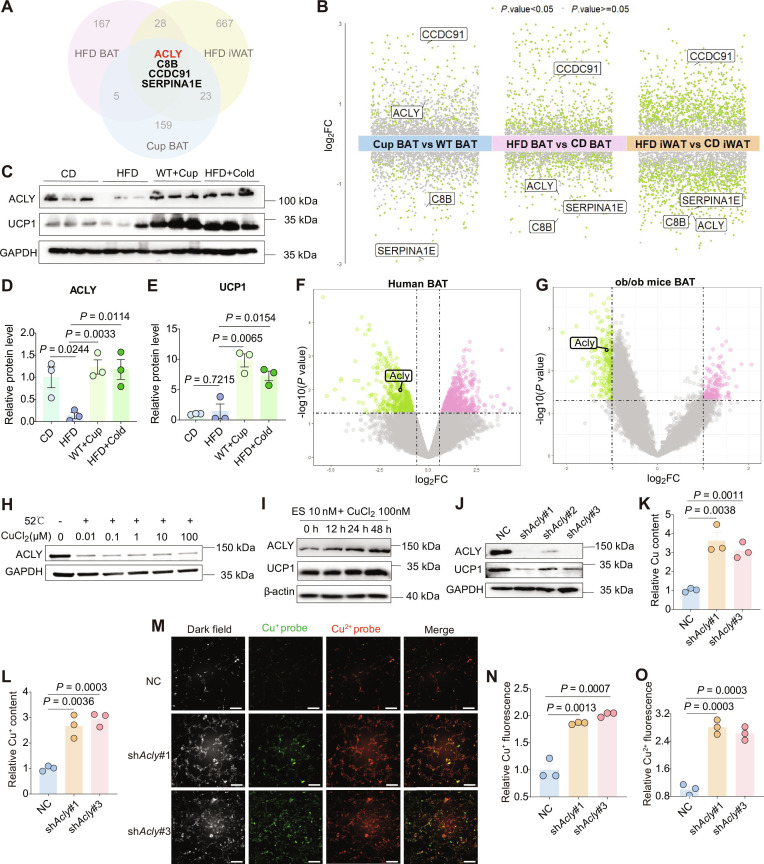
Down-regulation of ATP-citrate lyase (ACLY) causes copper overload in BAT. (A) Venn diagram showing the differentially expressed proteins (DEPs) in BAT (*n* = 6) and iWAT (*n* = 5) of DIO mice versus CD mice, as well as in BAT from Cuprizone (Cup)-treated mice (*n* = 3) versus CD mice BAT. (B) Multigroup volcano plots from proteomics analysis: DEPs in the BAT of DIO vs. CD mice, iWAT of DIO vs. CD mice, and BAT of Cup-treated mice vs. WT mice. The significance cutoff was set at *P* value < 0.05 and |Log2FC| > 1. (C to E) Western blot analysis and quantification for ACLY and UCP1 protein expressions in the BAT from CD, DIO, Cup-treated, and cold-exposed DIO groups. (F) Volcano plot of differentially expressed genes (DEGs) in the BAT of metabolic disorder patients versus healthy controls from the GSE220158 dataset. The significance cutoff was set at *P* value < 0.05 and |Log2FC| > 1. (G) Volcano plot of DEGs in the BAT of *ob*/*ob* mice versus CD mice from the GSE191009 dataset. The significance cutoff was set at *P* value < 0.05 and |Log2FC| > 1. (H) Cellular Thermal Shift Assay (CETSA) evaluating the binding capability between Cu^2+^ ions and ACLY proteins in lysate. (I) Analysis of ACLY and UCP1 protein expression in primary brown adipocytes treated with 10 nM Elesclomol (ES) and 100 nM CuCl^2^ for varying durations. (J) Evaluation of ACLY and UCP1 protein expression in *Acly*-knockdown C3H10T1/2 cells. (K and L) Relative total copper content and relative Cu^+^ content in *Acly*-knockdown C3H10T1/2 cells. (M) Fluorescence imaging of Cu^+^ and Cu^2+^ ions in *Acly*-knockdown C3H10T1/2 cells, which were detected using the respective fluorescent probes (scale bar: 20 μm). (N and O) Statistical analyses of Cu^2+^ and Cu^+^ levels in cells from various groups, *n* = 3. Data are presented as the mean ± SEM of independent biological replicates; *P* < 0.05 represents a statistically significant effect, while ns represents no significant effect.

Building upon our prior findings, we performed a proteomic analysis of the BAT and iWAT obtained from DIO mice, as well as the BAT from mice subjected to Cuprizone treatment. The corresponding tissues from the mice fed with a normal CD were also analyzed in parallel, serving as controls. By intersecting the differentially expressed proteins (DEPs) across these 3 experimental groups, we identified 4 common DEPs, including ACLY, C8B, CCDC91, and SERPINA1E (Fig. 2A). Notably, the ACLY protein, encoded by the *Acly* gene, exhibited a distinctive expression pattern among the 4 DEPs, characterized by a marked decrease in both the BAT and iWAT of DIO mice, while showing a significant elevation in the BAT of Cuprizone-treated mice (Fig. 2B). Further investigation through Western blot analysis revealed that ACLY and UCP1 exhibited analogous expression patterns. Both proteins were reduced in the BAT of DIO mice but increased in the BAT of Cuprizone-treated mice and cold-exposed DIO mice (Fig. 2C to E). These findings prompted us further to examine the role of ACLY in BAT copper homeostasis.

Furthermore, we analyzed 2 public datasets from the Gene Expression Omnibus (GEO) database, specifically the GSE220158 dataset [25] (mRNA-seq of BAT from patients with metabolic disorders treated with high-dose glucocorticoids versus healthy volunteers) and the GSE191009 dataset [26] (mRNA-seq of BAT from leptin-deficient *ob/ob* mice versus vehicle controls). Our analysis indicated a significant reduction in *ACLY* expression within the BAT of both metabolic disorder patients and *ob/ob* mice (Fig. 2F and G). Given the substantial increases in copper content observed in the BAT and iWAT of DIO mice, alongside a notable decrease in copper levels within the BAT of Cuprizone-treated mice, these findings imply a potential connection between ACLY expression, copper homeostasis, and thermogenic function in BAT.

To further explore the relationship between ACLY and copper homeostasis, we initially postulated that excess copper directly interacts with the ACLY protein, potentially resulting in its aggregation, a phenomenon noted during the copper ionophore-induced cell death (cuproptosis) for the lipoylated component of the TCA cycle [27]. Thus, we employed the Cellular Thermal Shift Assay (CETSA), a widely accepted method for the examination of protein engagement with ligand within a cellular environment [28]. Despite the application of high concentrations of Cu^2+^ (100 μM), our findings indicated that the thermal stability of the ACLY protein in cell lysates obtained from primary brown adipocytes remained largely unchanged, indicating that ACLY does not behave as a copper-binding protein (Fig. 2H). In addition, the cotreatment of primary brown adipocytes with ES and CuCl₂ resulted in an increasing trend in the ACLY and UCP1 protein levels (Fig. 2I), which was contrary to the observations made in vivo (Fig. 2C). These data suggest that copper overload alone is unlikely to account for the observed reduction in ACLY protein levels within the BAT of obese mice.

Next, we performed a knockdown of *Acly* expression in C3H10T1/2 cells using shRNA. All 3 sh*Acly* constructs resulted in a remarkable reduction of UCP1 expression, with sh*Acly*#1 and sh*Acly*#3 demonstrating the greatest efficacy (Fig. 2J). Therefore, our subsequent experiments concentrated on these 2 knockdown cell lines. Importantly, assessments of intracellular copper revealed that *Acly* knockdown led to significant increases in both the total copper content and the Cu^+^ level (Fig. 2K and L and Fig. S2A). In addition, fluorescent probing also revealed markedly elevated intracellular Cu^2+^ and Cu^+^ levels in *Acly*-knockdown cells (Fig. 2M to O). Collectively, these results suggest that ACLY plays a role in the regulation of copper homeostasis, likely by acting upstream to prevent copper overload.

### Down-regulation of ACLY induces copper overload via the NRF2/ATOX1 signaling cascade

**Fig. 3.**
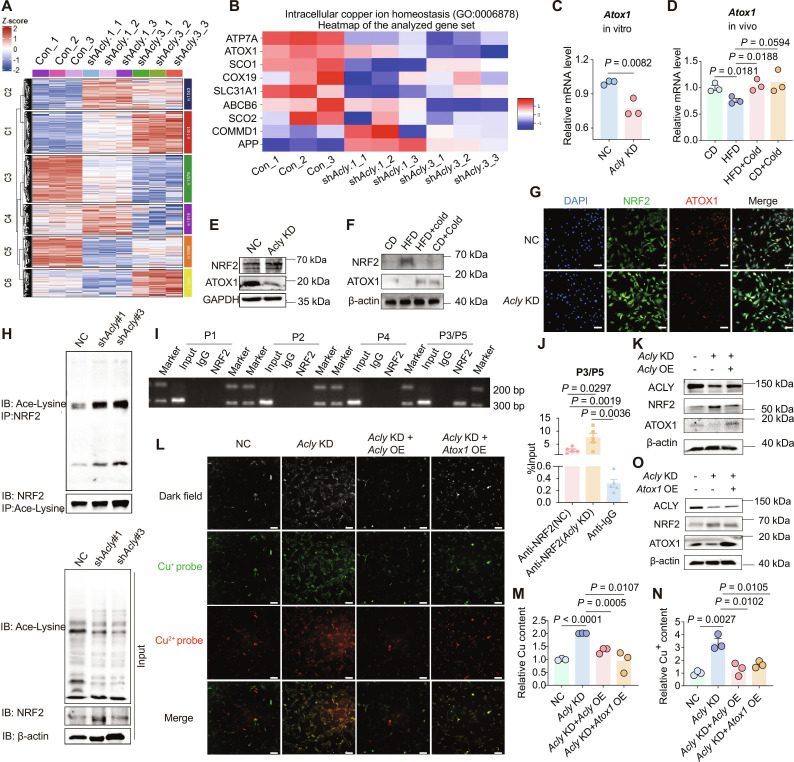
ACLY/NRF2/ATOX1 signaling pathway regulates copper homeostasis. (A) Heatmap illustration and clustering analysis of DEPs from the proteomics data of C3H10T1/2 cells with *Acly* knockdown (KD) and control (Con) cells. (B) Heatmap showing the expression profiles of proteins corresponding to the gene set of the “intracellular copper homeostasis” pathway. (C) Relative mRNA levels of *Atox1* in *Acly*-KD C3H10T1/2 cells, *n* = 3. (D) Relative mRNA levels of *Atox1* in the BAT of CD, DIO, cold-exposed DIO mice, and cold-exposed CD mice, respectively, *n* = 3. (E) Western blot showing the protein expressions of ATOX1 and NRF2 in *Acly*-KD cells. (F) Western blot showing protein expressions of NRF2 and ATOX1 in the BAT of CD, DIO, cold-exposed DIO mice, and cold-exposed CD mice, respectively. (G) Immunofluorescence staining images of ATOX1 and NRF2 in *Acly*-KD C3H10T1/2 cells (scale bar: 20 μm). (H) Immunoprecipitation assay assessing NRF2 acetylation in *Acly*-KD C3H10T1/2 cells. (I) ChIP assay confirming the binding of NRF2 to the P3/P5 region of the *Atox1* promoter. (J) ChIP-qPCR assay assessing the effect of *Acly* KD on NRF2 binding at the P3/P5 region of the *Atox1* promoter, *n* = 5. (K) Western blot showing the protein expressions of ACLY, NRF2, and ATOX1 in C3H10T1/2 cells with *Acly* KD followed by *Acly* overexpression (OE). (L) Fluorescence imaging of Cu^2+^ and Cu^+^ detected by fluorescent probes in cells under the following conditions: *Acly* KD followed by *Acly* OE, *Acly* KD followed by *Atox1* OE (scale bar: 20 μm). (M and N) Relative total copper content and relative Cu^+^ content in cells from various groups, *n* = 3. (O) Western blot showing the protein expressions of ACLY, NRF2, and ATOX1 in C3H10T1/2 cells with *Acly* KD followed by *Atox1* OE. Data are presented as the mean ± SEM of independent biological replicates; *P* < 0.05 represents a statistically significant effect, while ns represents no significant effect.

In light of the findings described above, we aimed to elucidate the molecular mechanism by which ACLY regulates copper homeostasis. First, we performed a proteomic analysis on C3H10T1/2 cells with *Acly* knockdown. DEPs from either one of the 2 *Acly*-knockdown cell lines were presented in a heatmap and clustered based on their expression profiles (Fig. 3A). From this, 4 clusters (C1, C2, C3, and C5) displaying consistent differential expression profiles were chosen, identifying 451 commonly up-regulated proteins and 434 commonly down-regulated proteins across the 2 sh*Acly* knockdown cell lines (Fig. S2B). Biological process enrichment analysis of these 985 DEPs revealed that *Acly* knockdown primarily affected biological processes related to “glucose and lipid metabolism”, “response to oxidative stress”, “regulation of metal ion transport”, and “response to metal ions” (Fig. S2C). Notably, within the biological function of “intracellular copper homeostasis”, a decrease in the expression of a key copper chaperone protein named antioxidant 1 copper chaperone (ATOX1) was observed following *Acly* knockdown (Fig. 3B). We should note that ATOX1 functions in maintaining copper homeostasis by delivering copper from the cytosol to copper ATPase proteins, and reduced ATOX1 expression can lead to intracellular copper overload [29].

We then examined the transcriptional levels of *Atox1* in both in vitro and in vivo models. We found that *Atox1* mRNA levels were significantly decreased not only in *Acly*-knockdown cells but also in the BAT of DIO mice (Fig. 3C and D). Conversely, *Atox1* transcription increased upon cold exposure in the BAT of DIO and CD mice (Fig. 3D). Given that ACLY is not a nuclear transcription factor, we reasoned that another nuclear transcription factor might mediate the transcriptional regulation of *Atox1* downstream of ACLY. Considering the close link between intracellular copper redox processes and oxidative stress responses and noting that the “response to oxidative stress” pathway was enriched in our proteomic analysis (Fig. S2C), we hypothesized that nuclear factor erythroid 2-related factor 2 (NRF2, encoded by *Nfe2l2*), a master regulator of oxidative stress and, crucially, a nuclear transcription factor, controls the transcription of *Atox1*.

Western blot analysis demonstrated a slight elevation in total NRF2 expression in *Acly*-knockdown cells, accompanied by a remarkable reduction in ATOX1 expression (Fig. 3E). This pattern was also conserved in the BAT of DIO mice (Fig. 3F). Furthermore, in response to cold exposure, NRF2 expression decreased while ATOX1 expression increased in both CD and DIO mice (Fig. 3F). Additionally, immunofluorescence analysis showed that the nuclear localization of NRF2 was significantly increased in the nuclear fraction of *Acly*-knockdown cells (Fig. 3G). Previous studies indicated that increased acetylation of NRF2 facilitates its nuclear translocation [30]. Thus, we examined NRF2 acetylation levels in *Acly*-knockdown cells and found a notable increase in NRF2 acetylation following *Acly* knockdown (Fig. 3H). In addition, we assessed the transcriptional levels of *Acly* and *Nfe2l2* in our models. No significant changes were detected in the mRNA levels of either *Acly* or *Nfe2l2* across the in vitro and in vivo conditions examined (Fig. S2D and E).

We hypothesized that NRF2 can transcriptionally suppress ATOX1 expression. To investigate whether NRF2 directly binds to the *Atox1* promoter, we used the JASPAR database to predict potential NRF2-binding sites within the *Atox1* promoter region, extending 2,000 bp upstream of the transcription start site (TSS). This analysis revealed 5 potential binding sites with a relative confidence score > 0.7 (Fig. S2F). Of note, 2 of the 5 sites (P3 and P5) largely share the binding sequences; thus, 4 corresponding primer sets were designed (Table S1). Subsequently, chromatin immunoprecipitation (ChIP) assays revealed a positive signal in the position P3/P5, located at positions −1,159 to −1,147 bp before the TSS (Fig. 3I). Furthermore, ChIP-quantitative PCR (qPCR) analysis demonstrated that down-regulation of ACLY enhanced the specific interaction between NRF2 and the *Atox1* promoter (Fig. 3J).

Next, we performed *Acly* overexpression in *Acly*-knockdown C3H10T1/2 cells. Restoration of ACLY protein expression rescued the phenotype: compared to *Acly*-knockdown alone, it decreased NRF2 expression, increased ATOX1 expression (Fig. 3K), and restored intracellular copper homeostasis (Fig. 3L to N and Fig. S2G and H). Next, we overexpressed *Atox1* in *Acly*-knockdown cells, which similarly reinstated intracellular copper homeostasis (Fig. 3L to N), without significantly affecting the expression of ACLY or NRF2 proteins (Fig. 3O).

In summary, these results indicate that down-regulation of ACLY likely enhances NRF2 acetylation, facilitating its translocation to the nucleus. In the nucleus, NRF2 represses *Atox1* transcription, resulting in reduced ATOX1 protein levels and ultimately contributing to copper overload.

### ACLY-mediated copper homeostasis regulated UCP1 expression through the SCO1/AMPK/PGC-1α pathway

**Fig. 4.**
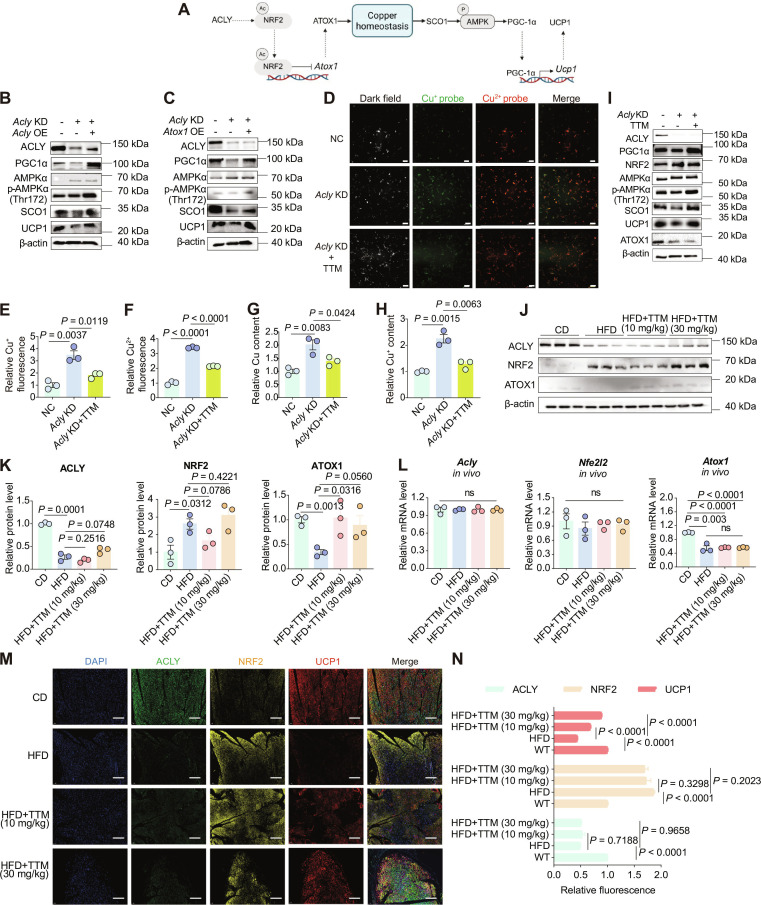
ACLY-mediated copper homeostasis regulates UCP1 expression through the SCO1/AMPK/PGC-1α pathway. (A) Scheme of the ACLY/NRF2/ATOX1 axis-regulated copper homeostasis influences UCP1 expression through the SCO1/AMPKα/PGC-1α pathway. (B and C) Western blot showing the protein expressions of ACLY, SCO1, AMPKα, p-AMPKα(Thr172), PGC-1α, and UCP1 in cells under the following conditions: *Acly* KD followed by *Acly* OE, *Acly* KD followed by *Atox1* OE. (D) Fluorescence imaging of Cu^2+^ and Cu^+^ detected by fluorescent probes in cells under *Acly* knockdown with TTM (10 μM) treatment (scale bar: 20 μm). (E and F) Statistical results of the relative fluorescence signal of Cu^2+^ and Cu^+^ in cells under *Acly* KD with TTM (10 μM) treatment, *n* = 3. (G and H) Relative total copper content and relative Cu^+^ content in cells under *Acly* KD with TTM (10 μM) treatment. (I) Western blot showing the protein expression of ACLY, NRF2, ATOX1, SCO1, AMPKα, p-AMPKα(Thr172), PGC-1α, and UCP1 in cells under *Acly* KD with TTM (10 μM) treatment. (J and K) Western blot analysis and quantification of ACLY, NRF2, and ATOX1 protein expressions in the BAT of TTM-treated DIO mice, *n* = 3. (L) Relative mRNA levels of *Acly*, *Nfe2l2*, and *Atox1* in the BAT of TTM-treated DIO mice, *n* = 3. (M and N) Immunofluorescence staining and semiquantification of ACLY, NRF2, and UCP1 relative expressions in the BAT of TTM-treated DIO mice, with the CD group used as reference, *n* = 3, (scale bar: 100 μm). Data are presented as the mean ± SEM of independent biological replicates; *P* < 0.05 represents a statistically significant effect, while ns represents no significant effect.

Next, we hypothesized that ACLY-mediated copper homeostasis (through the NRF2-ATOX1 axis) influences the expression of UCP1 through the SCO1/AMPK/PGC-1α pathway (Fig. 4A). To test this hypothesis, we first performed *Acly* overexpression in *Acly*-knockdown C3H10T1/2 cells. The restoration of ACLY protein expression increased the expression of SCO1, p-AMPKα(Thr172), PGC-1α, and UCP1 (Fig. 4B). Subsequently, overexpression of *Atox1* in *Acly*-knockdown cells also activated the SCO1/AMPK/PGC-1α pathway and increased UCP1 protein expression (Fig. 4C). Furthermore, we treated *Acly*-knockdown C3H10T1/2 cells with 10 μM TTM. TTM treatment likewise restored intracellular copper homeostasis (Fig. 4D to H), activated the SCO1/AMPK/PGC-1α signaling pathway, and increased UCP1 expression (Fig. 4I), without significantly altering NRF2 or ATOX1 expression (Fig. 4I).

To further substantiate that the ACLY/NRF2/ATOX1 axis operates upstream of copper homeostasis, we examined the protein and mRNA levels of NRF2 and ATOX1 in the BAT of TTM-treated DIO mice. TTM treatment did not significantly change the protein expression of ACLY or NRF2 (Fig. 4J). The observed variations of ATOX1 protein expression may result from the complicated regulation of cellular copper homeostasis (Fig. 4J and K). Importantly, TTM treatment had minimal effect on the mRNA levels of ACLY, NRF2, and ATOX1 (Fig. 4L). Additionally, immunofluorescence analysis also confirmed that TTM treatment effectively up-regulated UCP1 protein expression, while not significantly altering ACLY or NRF2 expression in BAT (Fig. 4M and N).

To further support our findings, we conducted mRNA expression correlation analyses of *ACLY* in BAT from patients with metabolic disorders (GSE220158), focusing on its association with thermogenesis and copper homeostasis-related genes (Fig. S3). The *ACLY* expression showed a significant positive correlation with the thermogenic marker *UCP1* (Fig. S3A and B). Furthermore, *ACLY* expression also exhibited significant positive correlations with the copper homeostasis-related genes *ATOX1* and *SCO1* (Fig. S3C and D). *ACLY* also showed a positive trend with *SCO2*, *SLC31A1*, and *CCS* (Fig. S3E, H, and M). These findings indicate that *ACLY* expression is closely linked to copper homeostasis and thermogenesis.

Taken together, these results demonstrate that ACLY protein expression modulates copper homeostasis in BAT via the NRF2/ATOX1 signaling axis, which subsequently influences UCP1 expression through the SCO1/AMPK/PGC-1α pathway.

### Discovery of SST as an upstream activator of ACLY

**Fig. 5.**
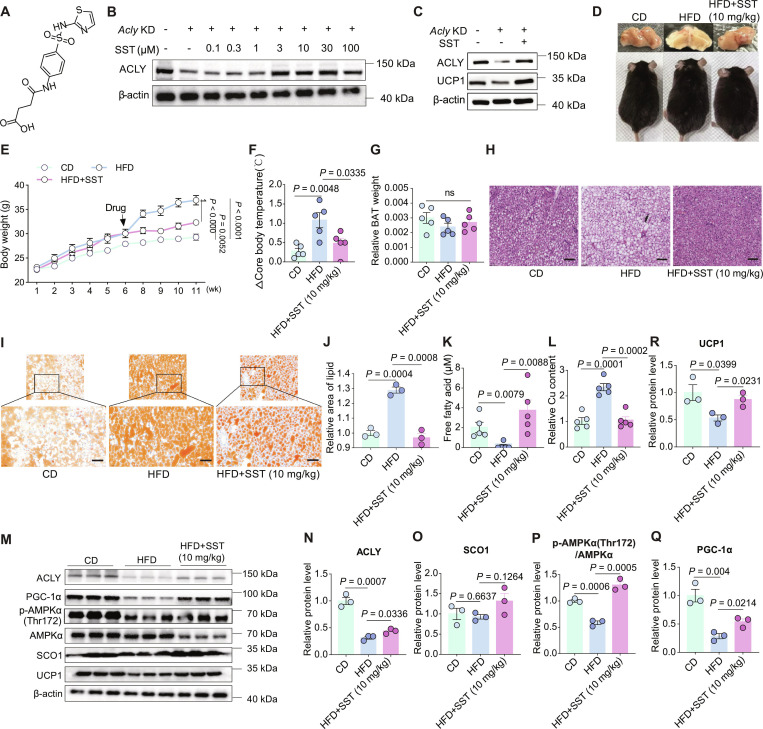
SST combats obesity and improves metabolism by promoting ACLY expression. (A) Chemical structure of succinylsulfathiazole (SST). (B) ACLY protein expression in *Acly*-knockdown C3H10T1/2 cells treated with different concentrations of SST for 24 h. (C) Protein expression of ACLY and UCP1 in *Acly*-knockdown C3H10T1/2 cells treated with 3 μM SST for 24 h. (D) Overall body morphology and BAT appearance of mice across the CD group, DIO group, and DIO group that received SST (10 mg/kg). (E) Changes in body weight among mice of the CD, DIO, and SST-treated groups, *n* = 6. Statistics for the overall effect represent the *P* values from a 2-way ANOVA followed by Dunnett’s test. (F) The absolute value of the decrease in core body temperature (ΔCore body temperature) after cold exposure for mice in each group, *n* = 5. (G) The ratio of BAT mass to body weight in mice of each group, *n* = 5. (H) H&E staining of BAT sections from mice in each group (scale bar: 20 μm). (I and J) Oil Red O staining of BAT sections from mice in each group, along with quantification (scale bar: 40 μm), *n* = 3. (K) Free fatty acid content in BAT of mice from each group, *n* = 5. (L) Relative copper content in BAT of mice from each group, *n* = 5. (M to R) Western blot analysis and quantification of ACLY, SCO1, p-AMPKα(Thr172), AMPKα, PGC-1α, and UCP1 protein expressions in the BAT of mice from each group, *n* = 3. Data are presented as the mean ± SEM of independent biological replicates; *P* < 0.05 represents a statistically significant effect, while ns represents no significant effect.

Despite the demonstrated effectiveness of TTM in alleviating copper overload in both cellular and murine models (Figs. 1 and 4), it is important to acknowledge that TTM has not yet received regulatory approval for clinical use, as the safety of this investigational compound following long-term usage remains undefined [31]. We reasoned that restoring ACLY expression levels may present a more effective strategy to normalize copper homeostasis and enhance BAT activity. Therefore, we utilized the Connectivity Map database (CMap, http://clue.io/cmap) to screen for small-molecule compounds capable of promoting ACLY expression based on proteomics data derived from *Acly*-knockdown cells. Among the compounds identified, SST (Fig. 5A), garcinol, and dapagliflozin exhibited the most significant effect on the overall proteome (Fig. S4A). We then examined whether these 3 compounds could augment ACLY expression using Western blot analysis. While none of these compounds affected ACLY protein expression in C3H10T1/2 cells, SST was found to significantly enhance ACLY protein expression in *Acly*-knockdown cells (Fig. S4B and C). This enhancing effect did not follow typical concentration dependence, as it became significant starting at a concentration of 3 μM, without compromising cell viability (Fig. S4D), and did not show further significant increase even at concentrations up to 100 μM (Fig. 5B).

Subsequently, we investigated the pharmacological effects of SST. We found that the treatment of SST (3 μM) for 24 h indeed increased the protein expression of UCP1 in *Acly*-knockdown cells (Fig. 5C). Administered via intraperitoneal injection at 10 mg/kg, SST attenuated the rate and extent of body weight gain induced by an HFD (Fig. 5D and E), counteracted the drop in body temperature due to cold exposure (Fig. 5F), unchanged BAT weight (Fig. 5G), suppressed the whitening of BAT (Fig. 5H and I), reduced lipid content in BAT (Fig. 5I and J), and increased FFA levels in BAT (Fig. 5K). Furthermore, SST treatment normalized the relative copper content within the BAT of DIO mice (Fig. 5L). SST treatment also increased ACLY protein expression in the BAT of DIO mice (Fig. 5M and N), and reinstated the SCO1/AMPK/PGC-1α signaling pathway (Fig. 5O to Q), leading to increased expression of UCP1 (Fig. 5R). Additionally, SST administration did not impact liver or kidney function in mice (Fig. S4E to G), but lowered blood glucose (Fig. S4H) and TG (Fig. S4I). While SST did not significantly impact T-CHO or LDL-C levels (Fig. S4J and K), it increased HDL-C levels in DIO mice (Fig. S4L). In addition, SST also decreased CK-MB levels (Fig. S4M).

These results collectively indicate that SST promotes ACLY protein expression, thereby restoring copper homeostasis and BAT activity in mice with metabolic disorders, ultimately resisting obesity and improving metabolic profiles.

### SST maintains BAT copper homeostasis and enhances UCP1 expression

**Fig. 6.**
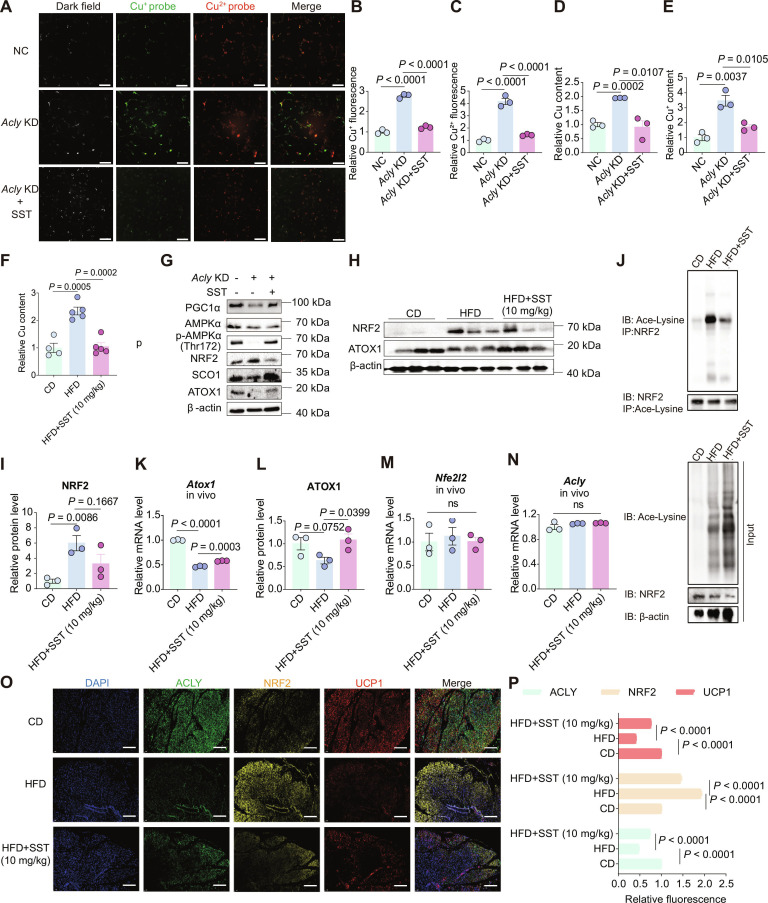
SST maintains copper homeostasis within BAT via NRF2/ATOX1 signaling axis, thereby enhancing the expression of UCP1 through the SCO1/AMPK/PGC-1α pathway. (A) Fluorescence imaging of intracellular Cu^2+^ and Cu^+^ detected by fluorescent probes after SST (3 μM) treatment in *Acly*-knockdown cells (scale bar: 20 μm). (B and C) Statistical analyses of relative intracellular Cu^2+^ and Cu^+^ levels after SST (3 μM) treatment in *Acly*-knockdown cells, *n* = 3. (D and E) Relative total copper content and relative Cu^+^ content in *Acly*-knockdown cells treated with SST (3 μM), *n* = 3. (F) Relative total copper content in the BAT of SST-treated (10 mg/kg) DIO mice, *n* = 3. (G) Western blot showing the protein expressions of NRF2, ATOX1, SCO1, AMPKα, p-AMPKα (Thr172), and PGC-1α in *Acly*-knockdown cells treated with SST (3 μM). (H and I) Western blot analysis and quantification for NRF2 in the BAT of SST-treated (10 mg/kg) DIO mice, *n* = 3. (J) Immunoprecipitation assay assessing NRF2 acetylation in the BAT of SST-treated (10 mg/kg) DIO mice. (K) Relative mRNA levels of *Atox1* in the BAT of SST-treated (10 mg/kg) DIO mice, *n* = 3. (L) Quantification results of ATOX1 in the BAT of SST-treated (10 mg/kg) DIO mice, *n* = 3. (M and N) Relative mRNA levels of *Acly* and *Nfe2l2* in the BAT of SST-treated (10 mg/kg) DIO mice, *n* = 3. (O and P) Immunofluorescence staining and semiquantification of ACLY, NRF2, and UCP1 relative expressions in the BAT of SST-treated (10 mg/kg) DIO mice, with the CD group used as reference, *n* = 3, (scale bar: 100 μm). Statistics for the overall effect represent the *P* values from a *t* test. *P* < 0.05 represents a significant effect, and ns represents no significant effect.

SST, functioning as an upstream agonist of ACLY, effectively mitigates copper overload and modulates relevant signaling pathways to restore BAT function. We next investigated the molecular mechanism underlying the pharmacological actions of SST. First, in the *Acly*-knockdown cells, SST successfully mitigated the copper overload induced by ACLY depletion (Fig. 6A to E). Consistently, in a parallel in vivo study, SST administration restored copper levels to normal within the BAT of DIO mice (Fig. 6F).

In vitro cell experiments demonstrated that SST reduced NRF2 protein expression, increased ATOX1 expression, and activated the SCO1/AMPK/PGC-1α pathway, which ultimately led to an elevation of UCP1 protein expression in the *Acly*-knockdown cells (Fig. 6G). Similarly, in DIO mice, SST reduced both NRF2 protein expression (Fig. 6H and I) and its acetylation levels in BAT (Fig. 6J), while also enhancing *Atox1* transcription (Fig. 6K), resulting in elevated ATOX1 protein levels (Fig. 6G and L). Notably, while SST enhanced *Atox1* transcription due to the suppressed acetylation of NRF2 levels (Fig. 6K), it did not significantly affect the transcriptional levels of *Nfe2l2* (Fig. 6M).

**Fig. 7.**
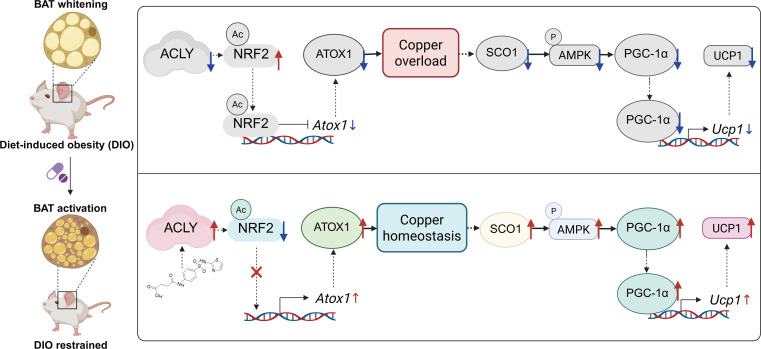
Proposed mechanism for the role of ACLY in BAT copper homeostasis and thermogenesis. The down-regulation of ACLY impairs BAT activity by inducing copper overload (top panel). In obese mice with metabolic abnormalities, reduced ACLY expression leads to an increase in the acetylation of the transcription factor NRF2, which enhances its localization in the nucleus. As a result, NRF2 interacts with the promoter of *Atox1*, suppressing its transcription and leading to decreased ATOX1 expression. The depletion of ATOX1 results in copper overload in brown adipocytes. Elevated copper levels lead to a reduction in SCO1 expression, which decreases AMPK phosphorylation. This ultimately impairs the transcriptional regulation of *Ucp1* by the nuclear transcription factor PGC-1α, resulting in diminished UCP1 expression and compromised BAT function. Conversely, reinstating ACLY protein expression counteracts BAT whitening in obese mice by re-establishing copper homeostasis, which improves systemic glucose and lipid metabolism (bottom panel). SST treatment of obese mice promotes ACLY expression and restores ACLY protein levels, leading to a decrease in NRF2 acetylation. This reduction allows for normal transcription and expression of ATOX1, thereby maintaining copper homeostasis in brown adipocytes. Consequently, the SCO1/AMPK/PGC-1α pathway is restored, facilitating the recovery of UCP1 expression. Collectively, these changes help suppress the whitening of BAT, preserving its critical role in systemic glucose and lipid metabolism. Created with BioRender.com.

In addition, SST also hardly affected the mRNA level of *Acly* (Fig. 6N). The influence of SST on ACLY protein expression was limited to *Acly*-knockdown cells in vitro, with no discernible impact observed in control cells (Fig. S4B). Considering its lack of effect on *Acly* mRNA, SST is unlikely to regulate ACLY at the transcriptional level and may instead act through a posttranslational mechanism. We therefore assessed the ubiquitination status of ACLY proteins. Immunoprecipitation with an ACLY antibody revealed that SST effectively reduces ACLY ubiquitination, suggesting that SST may stabilize ACLY by suppressing its degradation through the ubiquitin–proteasome pathway (Fig. S4N). Finally, immunofluorescence analysis further confirmed that SST increased ACLY protein expression, decreased NRF2 expression, and ultimately restored the expression of the key thermogenic protein UCP1 (Fig. 6O and P). This process maintains copper homeostasis in BAT via the NRF2/ATOX1 signaling axis, thereby regulating BAT activity through the SCO1/AMPK/PGC-1α pathway to combat obesity and ameliorate metabolic disorders (Fig. 7).

## Discussion

Obesity and its associated metabolic disorders are becoming increasingly prevalent in humans. BAT is a metabolically active tissue that plays important roles in regulating body metabolism and homeostasis. Chronic obesity may lead to the whitening of BAT and its impaired function [32], which, in turn, can exacerbate obesity and metabolic disorders [33]. Therefore, elucidating the pathological mechanisms underlying reduced BAT’s function and restoring or maintaining BAT activity may restrain obesity and ameliorate systemic metabolic dysregulation [34]. In recent years, the role of metal ion homeostasis in various diseases has attracted increasing attention [35]. However, the mechanisms by which copper homeostasis regulates the activity of BAT and its specific actions in the context of obesity-associated metabolic disorders remain poorly understood. It is important to note that, because of the anatomical location of BAT in humans and the challenges associated with obtaining BAT biopsies [36], there has been a lack of direct measurement data on the modulation of copper content in human BAT across different physiological conditions to date.

This study provides the first evidence of copper overload in the BAT of DIO mice. Interventions such as copper chelator and cold exposure successfully normalized copper levels, reactivated UCP1 expression, attenuated BAT whitening, and enhanced overall metabolic health. Proteomic profiling revealed decreased ACLY expression in settings of copper excess. Further mechanistic studies showed that ACLY down-regulation led to increased acetylation of NRF2 and its nuclear translocation, which transcriptionally suppresses ATOX1, aggravating copper accumulation and consequent BAT functional impairment. Using CMap screening, we discovered SST as a novel, effective compound that promotes ACLY expression. Administration of SST successfully reversed ACLY down-regulation, ameliorated copper metabolism defects, restored BAT functionality, effectively mitigated weight gain, and improved metabolic parameters. These results elucidate a previously unknown pathway contributing to obesity-related BAT dysfunction and nominate ACLY as a potential target for therapeutic intervention, with SST serving as a candidate for drug repurposing in metabolic syndromes.

ACLY is a crucial enzyme involved in de novo lipogenesis (DNL), mainly through the production of acetyl-CoA in the cytosol. The function of ACLY has been primarily studied in the liver. However, its role in BAT remains unclear. Most research has concentrated on examining its role in hepatic lipogenesis, indicating its potential as a therapeutic target for hyperlipidemia, with existing ACLY-targeted drugs, such as the Food and Drug Administration-approved bempedoic acid [37], primarily acting as inhibitors [38]. However, recent findings by Yenilmez et al. [39] showed that the hepatocyte-selective depletion of ACLY in obese mice paradoxically increased the total DNL flux, while Liu et al. [40] revealed that bempedoic acid operates independently of ACLY and that ACLY deficiency unexpectedly exacerbates diet-induced steatosis. These studies collectively suggest that hepatic ACLY may play an important role in suppressing diet-induced lipid accumulation. Thus, there is a necessity to reassess the role and function of ACLY, particularly concerning its mechanisms across various tissues and organs. Very recently, a cross-tissue proteomic atlas concerning cold stimulation indicated that the protein expression levels of ACLY, ATOX1, SCO1, and UCP1 in the BAT of mice were increased following cold exposure, corroborating our experimental findings (Fig. S5). Moreover, it is noteworthy that under cold stimulation, ACLY expression in BAT contrasts with its expression in the liver, where a down-regulation was observed. This observation further indicates that ACLY may be differentially regulated or serve different roles across different tissues (Fig. S5). Korobkina et al. [41] have recently provided the first evidence of the critical role of ACLY in coupling fatty acid synthesis and oxidation in BAT, demonstrating that ACLY deficiency can lead to an overload of the TCA cycle, trigger integrated stress responses, and impair the thermogenic function of UCP1. While Korobkina’s investigation was confined to examining ACLY’s role in BAT using normal chow-fed mice, our study employs a markedly different approach by investigating an HFD-induced obesity model to uncover a novel mechanism for ACLY in BAT under conditions of excess energy.

Our work demonstrated that the decline of ACLY in DIO mice triggered copper overload. For now, the upstream signals that trigger the initial decline of ACLY in DIO mice remain elusive. It is possible that the adipocyte initially modulates the expression of ACLY in an effort to recalibrate energy homeostasis or copper sensing in response to the metabolic stress associated with an HFD [38]. The data presented suggest that even if the initial copper overload response begins as compensatory, it may ultimately evolve into a pathological state, supported by the following evidence: (a) Functional restoration via copper chelation. Treatment with the copper chelator TTM in DIO mice significantly enhanced UCP1 expression, reversed BAT whitening, and improved systemic metabolic parameters (Fig. 1D to Q). This functional restoration upon copper reduction indicates that the copper excess was detrimental. (b) Mechanistic suppression of thermogenic pathways. Our data demonstrate that copper overload in DIO mice leads to the suppression of the SCO1/AMPK/PGC-1α pathway, which is essential for UCP1-mediated thermogenesis (Fig. 1M to P). Importantly, a reduction in copper levels reinstated this pathway.

In our preliminary exploration, Cuprizone, a copper chelator capable of crossing the blood–brain barrier, was utilized; however, its ability to bind multiple metals renders it nonspecific in manipulating copper levels in mice [42,43]. In contrast, TTM is recognized as a highly potent systemic copper chelator with a generally favorable safety profile [31]. Nevertheless, it is important to acknowledge that the biological effects of TTM extend beyond simple copper removal, as systemic administration via gavage may produce off-target effects in other organs that also contribute to systemic metabolism. Currently, the lack of BAT-specific copper chelators presents a technical challenge in the field. The future development of targeted delivery systems will be essential to minimize off-target effects and further validate the tissue-specific role of copper homeostasis in regulating BAT activity.

While copper chelation alone was sufficient to partially restore BAT copper levels and UCP1 expression in DIO mice via the SCO1/AMPK/PGC-1α pathway, we reasoned that the absence of reliable and specific biomarkers to precisely evaluate the therapeutic efficacy and adjust the dosage of copper chelators may hinder the translational application of this direct copper-modulatory strategy in humans. Alternatively, regulating copper homeostasis imbalance through modulating copper metabolic pathways could provide a safer and more effective therapeutic strategy for diseases characterized by disrupted copper homeostasis [13]. In this study, we proposed and demonstrated that targeting or restoring the upstream regulator ACLY is essential for reversing the molecular mechanisms of copper overload, which involve elevated acetylation and nuclear localization of NRF2, as well as suppressed expression of ATOX1, and their downstream metabolic consequences.

A significant discovery in this study is the identification of SST as the first pharmacologically relevant compound capable of enhancing ACLY expression, thereby restoring copper homeostasis and thermogenic activity in both the ACLY-deficient cell model and DIO mice. Mechanistically, our data indicated that SST may stabilize ACLY by suppressing its degradation through the ubiquitin–proteasome pathway (Fig. S4N), although the specific type and site of ubiquitin linkage on ACLY remain unclear. In addition, the precise molecular mechanisms through which SST affects ACLY ubiquitination and degradation require further investigation. Interestingly, the dose–response curve for SST shows no clear concentration dependence above 3 μM (Fig. 5B), suggesting that this inhibitory capacity on the ubiquitin-mediated degradation may have reached saturation around this drug concentration. Originally used clinically as a sulfonamide antibiotic, our investigation explores the potential repurposing of SST for modulating BAT function. The data indicate that SST possesses an excellent potency and safety profile, suggesting that repurposing SST, or the development of agents mimicking its ACLY-boosting mechanism, could represent a promising therapeutic strategy for obesity-associated metabolic disorders. Although our data support that SST can directly enhance the expression of ACLY within adipocytes (Fig. 5B), it is important to note that, given its antibiotic properties, the possibility remains that SST might exert effects by modulating gut microbiota [44]. Future studies should focus on the design of new SST derivatives or formulations as targeted therapy for BAT, potentially through nanotechnology, to yield more definitive outcomes.

In conclusion, our findings suggest that ACLY plays a previously unrecognized, critical role in maintaining copper homeostasis to uphold thermogenic function, providing a novel perspective on the role of ACLY in BAT under conditions of obesity: restoring normal ACLY expression can re-establish copper ion homeostasis in BAT, reverse its “whitening”, enhance BAT functional activity, and ultimately lead to significant improvement in metabolic disorders.

## Materials and Methods

### Mice experiments

Male C57BL/6J mice (18 to 20 g) were purchased from GemPharmatech Co., Ltd. Upon arrival, mice were acclimated to the animal facility for 1 week and maintained under controlled environmental conditions at 25 °C. Starting at 8 weeks of age, these mice were fed an HFD (XTHF60, XIETONG SHENGWU, XTM04-001) to establish a model of obesity. During the period of HFD feeding, the body weight was measured every 5 d. For the TTM-treated group, TTM was dissolved in double-distilled water and administered via intragastric gavage [45] at doses of 10 and 30 mg/kg, respectively. For the SST-treated group, SST was dissolved in normal saline, and the pH was adjusted to approximately 7.0 using 1 M NaOH, yielding a clear, transparent solution without precipitation. SST was then administered via intraperitoneal injection at a dosage of 10 mg/kg. All mouse-related experiments were conducted in strict compliance with the *Guide for the Care and Use of Laboratory Animals* and approved by the Laboratory Animal Ethics Committee of the First Affiliated Hospital of Southern University of Science and Technology (Registration No. AUP-240226-LX-0069-01).

### Cell culture

HEK293T/17 cells were cultured in DMEM (Dulbecco’s Modified Eagle Medium, Procell system, PM150410) supplemented with 10% fetal bovine serum (FBS) and 1% penicillin/streptomycin (P/S). C3H10T1/2 cells were cultured in MEM (minimum essential medium, Procell system, PM150410) supplemented with 10% FBS and 1% P/S. All cell models were regularly tested for mycoplasma contamination. Cells were passaged every 2 d using 0.25% trypsin and 0.02% EDTA for dissociation. All cells were passed fewer than 20 times for experiments. Culture conditions were maintained as follows: 5% CO₂, 37 °C, and 70% to 80% humidity in the incubator. The freezing medium was composed of 90% serum and 10% DMSO (dimethyl sulfoxide), which was prepared fresh immediately before use. Primary brown adipocytes were extracted and cultured using established methods [46]. Following anesthesia, C57BL/6J mice were immersed in 75% ethanol for 5 min. BAT was excised from the scapular region and then placed in precooled phosphate-buffered saline (PBS). The BAT was cleaned of white adipose tissue and connective tissue, minced, and digested with Collagenase II at 37 °C for 30 min. After digestion, the tissue homogenate was filtered through a 70-mesh nylon sieve, and the filtrate was centrifuged to remove the supernatant. The pellet obtained was resuspended in red blood cell lysis buffer, allowed to stand, centrifuged again, washed, and then resuspended. The isolated preadipocytes were plated in DMEM and cultured until reaching 80% to 90% confluence, at which point the medium was replaced with adipocyte induction cocktail I (DMEM + 10% FBS + 0.5 mM 3-isobutyl-1-methylxanthine + 1 μM rosiglitazone + 1 μM dexamethasone + 1 nM T3 + 850 nM insulin) for 48 h, followed by replacement with adipocyte induction cocktail II (DMEM + 10% FBS + 0.5 μg/ml insulin) for another 48 h. Finally, the cells were maintained in complete DMEM until differentiation into mature adipocytes that were filled with lipid droplets.

### Cold challenge experiments

In the acute cold exposure experiment, mice were given access to only drinking water, with no food provided, starting from the night before the experiment. On the experimental day, cages without bedding were precooled for 1 h in a 4 °C experimental animal refrigerator. Mice from each group were then transferred to these precooled cages, where they received precooled drinking water without food. Rectal temperature measurements were taken hourly using a probe, with 2 mice housed per cage (RET-3, ThermoWorks). After cold exposure, the mice were returned to room temperature housing and given adequate food and water.

In the repeated subacute cold exposure experiment, mice were fasted from food and water for 6 h prior to the experiment. On the experimental day, cages without bedding were precooled for 1 h in a 4 °C experimental animal refrigerator. Then, mice from each group were transferred to these precooled cages, with only 2 mice per cage, and no food or water was provided. After 1 h of cold exposure, the mice were returned to their housings at RT and given adequate food and water. The cold exposure regimen was repeated continuously for a period of 7 d.

### Tissue collection and processing

As previously described [46], after anesthetizing the mice with isoflurane, orbital blood was collected. The collected whole blood was aliquoted for plasma and serum preparation, respectively. Subsequently, the mice were euthanized by cervical dislocation, after which BAT was isolated. A portion of the BAT was snap-frozen in liquid nitrogen for subsequent analyses, including Oil Red O staining and Western blotting. Another portion was fixed with 4% paraformaldehyde (PFA) for hematoxylin–eosin (H&E) staining and immunofluorescence staining. The remaining tissue was used for the measurement of copper content and FFA content.

### H&E staining

Mouse BAT was collected and fixed in 4% PFA solution for 24 h. Following fixation, the samples underwent a series of processes including dehydration, clearing, embedding, sectioning, and dewaxing. The sections were then immersed in hematoxylin staining solution for 5 min to stain the cell nuclei. Subsequently, they were rinsed with double-distilled water (5 min), briefly dipped in 1% hydrochloric acid ethanol (3 s), rinsed under tap water (20 min), and finally blued with 0.6% ammonia water. Next, the sections were immersed in double-distilled water for 2 min, followed by eosin staining solution for 3 min to stain the cytoplasm. The sections were dehydrated and cleared again, then mounted with neutral balsam. The morphological characteristics of the tissue were examined under a microscope.

### Oil red O staining

Following the Optimal Cutting Temperature (OCT) embedding and sectioning, BAT sections were brought to RT, fixed, rinsed with tap water, and air-dried. Saturated ORO staining solution (Servicebio, G1015) was thoroughly mixed with distilled water at a 3:2 ratio and incubated at 4 °C overnight. The mixture was then filtered once using filter paper, stored at 4 °C for an additional 24 h, and filtered a second time to prepare the ORO working solution. The sections were immersed in the ORO staining solution for 8 to 10 min (covered to avoid light). After removal, the sections were left for 3 s before being sequentially transferred to 60% isopropanol for differentiation. The sections were then sequentially rinsed in distilled water. Next, the sections were subjected to immersion in hematoxylin for counterstaining, followed by rinsing with distilled water. After incubation with a differentiation solution, the sections were rinsed with water, blue-stained with a bluing reagent for 1 s, reimmersed in distilled water, and finally photographed and scanned.

### Intracellular copper fluorescence detection

To assess Cu^2+^ fluorescence, cells were treated with 10 μM Rhodamine β-hydrazide for 24 h, followed by measurement at λ_ex_/λ_em_ = 532/588 nm. For Cu^+^ detection, cells were treated with 3 μM BioTracker Green Copper Live Cell Stain for 24 h and with measurement at λ_ex_/λ_em_ = 488/507 nm. Both fluorescent dyes were added to the cells concurrently. After the incubation period, cells were examined and photographed using a fluorescence microscope. The acquired images were then analyzed and quantified with ImageJ software.

### Copper content detection assays

For tissue samples, the Cu^2+^ content was measured using a Copper (Cu^2+^) Colorimetric Assay Kit (Elabscience, E-BC-K300-M). According to the instructions, tissues were homogenized in double-distilled water, and centrifugation was performed to collect the supernatant. The supernatant was divided for analysis, with one portion reserved for protein concentration measurement, while a 15-μl aliquot was used to determine the optical density (OD) value at 580 nm. The relative copper content was calculated based on the standard curve, normalized to the protein concentration. For cell samples, measurements were conducted using 2 kits: the Cellular Cuprous Fluorometric Assay Kit (Elabscience, E-BC-F102) for Cu^+^ content and the Cell Copper (Cu^2+^) Colorimetric Assay Kit (Elabscience, E-BC-K775-M) for Cu^2+^ content. As per the kit instructions, cells were lysed with the corresponding solvent, followed by centrifugation, quantification of protein concentration, and determination of copper content. The relative copper content was similarly calculated, normalized to protein concentration according to the standard curve.

### Inductively coupled plasma mass spectrometry

Following the lysis of cells or adipose tissue using PBS containing protease inhibitors and the determination of protein concentration, concentrated nitric acid and 30% hydrogen peroxide were added to the mixture. The resulting solution was sealed and subjected to microwave-assisted acid digestion in a microwave digestion system. Upon the completion of the digestion process, the sample was allowed to cool and was subsequently diluted to a final volume of 5 to 10 ml with ultrapure water. The samples were analyzed on inductively coupled plasma mass spectrometry (Agilent 7700), with an internal standard method implemented to correct for matrix effects and instrument drift. The data obtained were reported in terms of μg/g protein content or tissue weight.

### FFA content detection

In accordance with the manufacturer’s protocol (Beyotime, S0215M), tissue samples (10 to 20 mg) were homogenized in 100 to 200 μl of BeyoLysis Buffer A for Metabolic Assay at 4 °C. The resulting homogenate was centrifuged at 12,000 *g* for 5 min at 4 °C, and the supernatant was collected for analysis. A cholesterol standard solution was diluted to various concentrations to create a standard curve. A working solution for FFA detection was prepared, and the sample fluorescence intensity was measured using a multifunctional microplate reader (λ_ex_/λe_m_ = 560/590 nm). A standard curve was constructed and used to calculate sample concentrations, which were adjusted for protein content.

### Mass spectrometry-based proteomic analysis

The sample preparation for proteomic analysis was carried out as previously described [47,48]. Tissue and cell samples were thawed on ice after removal from the −80 °C freezer. Subsequently, lysis buffer, which contains100 mM triethylammonium bicarbonate (TEAB), 8 M urea, and 1% sodium deoxycholate, was added to homogenize the tissues or cells. Following centrifugation at 12,000 *g* for 15 min, the supernatant was harvested. Following protein concentration measurement, the samples were diluted to 1 μg/μl with lysis buffer. Then, 20 mM DTT and 40 mM IAA were added; the mixtures were vortexed and briefly centrifuged, and finally incubated at RT in the dark for 30 min. The TCA solution was introduced and incubated at −20 °C for >1 h. Postincubation, the samples were centrifuged, and the supernatant was discarded. Prechilled acetone was added, followed by vortexing and centrifugation, after which the supernatant was discarded. Samples were then air-dried. Next, TEAB buffer, LysC, and trypsin were added in sequence, and the samples were incubated overnight at 37 °C. Following desalting, the peptide concentrations were measured using Nanodrop, and proceeded to LC-MS/MS measurement in a data-independent acquisition (DIA) mode. Data analysis was performed using the following software: DIA-NN (v.1.8.0) [49] for spectral processing and quantification, and gene set enrichment analysis [50] for functional enrichment analysis.

### Immunoprecipitation assay

Cells were collected and subjected to centrifugation at 500 *g* for 5 min at 4 °C, after which the pellet was washed twice with ice-cold 1× PBS. For the purpose of lysis, the pellet was resuspended in 500 μl of lysis buffer (50 mM Tris, 150 mM NaCl, 1% NP-40, and 1 mM EDTA, pH 7.2) supplemented with protease inhibitors and incubated on ice for 30 min. For tissue samples, homogenization was performed directly in the supplemented lysis buffer, followed by centrifugation at 10,000 *g* for 30 min at 4 °C to collect the supernatant. A small aliquot of each lysate supernatant was saved as the Input control. The remainder was incubated with 2 to 10 μg of primary antibody on a rotator at 4 °C overnight. Following this, 20 μl of protein A/G beads was added, and incubation continued for an additional 4 h at 4 °C. The beads were then pelleted by centrifugation at 3,000 *g* for 5 min at 4 °C and were washed 3 to 4 times with 500 μl of lysis buffer. The antigen–antibody complexes were eluted using 50 μl of elution buffer to isolate the target protein, which was then mixed with loading buffer and boiled at 100 °C for 15 min prior to sodium dodecyl sulfate–polyacrylamide gel electrophoresis (SDS-PAGE) electrophoresis.

### ChIP-qPCR assay

The assay was carried out using the BeyoChIP Enzymatic ChIP Assay Kit (Beyotime, P2083S), following the manufacturer’s instructions. Cell samples were cross-linked with formaldehyde to stabilize protein–DNA complexes, and the cross-linking was subsequently terminated with glycine. Following this, the cells were lysed, and the nuclei were prepared. Micrococcal nuclease (MNase) was used to enzymatically fragment the chromatin into sizes ranging from 1 to 5 nucleosomes (approximately 150 to 900 bp), followed by sonication to disrupt the nuclear membrane and release the chromatin fragments. A small portion of the sample was saved as the Input control. The remaining sample was treated with a target protein-specific antibody and incubated overnight at 4 °C. Protein A/G magnetic beads were then used to enrich the antibody–protein–DNA complexes. The beads were subjected to sequential washes with low salt, high salt, LiCl, and TE buffers to remove nonspecific binding. The complexes were subsequently eluted and heated at 65 °C to reverse the cross-links, followed by digestion with proteinase K. Final DNA purification was achieved through phenol–chloroform extraction and ethanol precipitation. The purified DNA can be used for qPCR analysis to detect target gene sequences. Enrichment of the target sequences was evaluated using the % Input method.

### Cell transfection

Stable knockdown of *Acly* in C3H10T1/2 cells was achieved via the application of lentiviral shRNA. shRNAs targeting the genes of interest were constructed by cloning the designated sequences into the pLKO.1 vector. Virus production involved cotransfection in HEK293T/17 cells with the constructed vector and the packaging plasmids psPAX2 and pMD2.G. Next, C3H10T1/2 cells were transduced with the resulting lentivirus supernatant for 24 h, followed by selection with puromycin (2 μg/ml) for 5 to 7 d. The reduction in target protein levels was confirmed through Western blot analysis. The sequences of shRNA are listed in Table S1. For the establishment of stable overexpression cell lines, the coding sequences of *Acly* and *Atox1* were retrieved from the NCBI database and cloned into the pLVX vector, which imparts G418 resistance. ACLY and ATOX1 levels were similarly analyzed by Western blotting. All other steps followed the previously described protocol.

### Immunofluorescence staining (in vitro)

Logarithmic-phase cells were seeded into 24-well plates by adding 2.0 × 10^4^ cells to each well. The cells were allowed to adhere and incubated for 24 h, followed by 2 washes with PBS and fixation using 500 μl of immunostaining fixative at RT for 15 min. After another 2 washes with PBS, the cells were incubated with 500 μl immunostaining wash buffer for 30 min, washed again with PBS, and then blocked with 500 μl of immunostaining blocking buffer at RT for 30 min. Primary antibodies (anti-NRF2 and anti-ATOX1) were diluted in PBS (100 μl) and applied to the cells, which were then incubated at 4 °C for 18 to 24 h. Following 2 washes with PBS, species-matched fluorescent secondary antibodies were added and incubated in the dark at RT for 1 h. After 2 further washes with PBS in the dark, samples were stained with 4′,6-diamidino-2-phenylindole (DAPI) (5 μg/ml, 100 μl) in the dark for 15 to 20 min. A final PBS wash was performed prior to imaging via fluorescence microscopy. The fluorescence intensity was quantified under identical microscope settings. ImageJ software was utilized to convert the images to grayscale, and the mean fluorescence intensity of the positive regions was measured after applying a consistent threshold.

### Multiplex immunofluorescence (ex vivo)

For fixed BAT sections, heat-induced antigen retrieval was carried out in a citric acid antigen-retrieval solution (pH 6.0, Servicebio G1202) for 30 min, followed by 3 5-min washes with PBS. After circumscribing and treating with a hydrogen peroxide solution, the tissue sections were blocked using 10% rabbit serum (Servicebio G1209) in a humid chamber for 30 min at RT. Sequential multiplex immunofluorescence was then performed. The sections were incubated overnight at 4 °C with ACLY primary antibody (HUABIO ET1609-37; 1:1,000 in PBS). After washing with PBS, a secondary antibody (Servicebio GB23303) was applied at RT for 50 min. Subsequently, iF555-Tyramide (Servicebio G1233; 1:500; λ_ex_/λ_em_ = 557/570 nm) was introduced in the dark at RT for 10 min. Following a wash with TBST, the sections were treated with an antibody elution buffer (Servicebio G1266) (RT for 5 min, then 37 °C for 30 min) and finally rinsed 3 times with TBST. This sequence of 10% serum blocking, primary antibody, secondary antibody, tyramide signal amplification, elution, and washes was then repeated for the UCP1 antibody (Proteintech 23673-1-AP, 1:2,000) using iF488-Tyramide (Servicebio G1231, 1:500; Ex/Em 491/516 nm), and finally for NRF2 antibody (Servicebio GB113808, 1:5,000) using iF647-Tyramide (Servicebio G1232, 1:500; λ_ex_/λ_em_ = 656/670 nm). Before mounting and imaging, the final sequential steps included nuclear counterstaining with DAPI and quenching of tissue autofluorescence.

### Cellular thermal shift assay

Protein–ligand engagement was assessed using the CETSA method according to established protocols [51]. For the experiments involving cell lysates, primary brown adipocyte cell lysates were prepared and then incubated for 10 min with varying concentrations of CuCl₂ or DMSO. Following incubation, the samples were heated at 52 °C for 3 min and centrifuged at 10,000 *g* for 30 min at 4 °C to remove insoluble material. The resulting clarified supernatant was then subjected to SDS-PAGE, and the proteins of interest were detected by Western blotting.

### Immunoblotting

Protein samples obtained from adipose tissue or cells were analyzed by immunoblotting using an adapted protocol [52]. Cells or tissues were lysed in radioimmunoprecipitation assay (RIPA) buffer (Beyotime, P0013B) containing a protease and phosphatase inhibitor cocktail (Thermo Fisher Scientific, 78442). Protein concentrations were determined using the BCA Protein Assay Kit (Thermo Fisher Scientific, 23225). Equal protein amounts per sample were then subjected to separation by SDS-PAGE. Primary antibodies were incubated at 4 °C overnight, followed by a 1-h incubation at RT using species-appropriate secondary antibodies. After the reaction with the ECL substrate, enhanced chemiluminescence signal detection was performed using the G: BOX Chemi XX9 (Syngene).

### Quantitative reverse transcription polymerase chain reaction

RNA isolation was performed using TRIzol reagent (Life Technologies, 15596). Reverse transcription was then conducted with ABScript III RT Mix (ABclonal, M21478) to generate cDNA. Next, qPCR was carried out using TB Green Fast qPCR Mix (Takara, RR430A) on a real-time PCR system. Gene expression was normalized to GAPDH, and all primer sequences are provided in Table S1.

### Statistical analysis

Statistical analyses were conducted with GraphPad Prism 8.0. Data are presented as mean ± SEM unless specified. Comparisons between 2 groups were made using Student *t* test, while multiple comparisons were assessed by ordinary 2-way analysis of variance (ANOVA).

## Data Availability

All proteomics results from this study have been uploaded to the ProteomeXchange database (https://www.proteomexchange.org/), with data accession numbers PXD067824, PXD067834 , PXD067866, and PXD067877. The GSE220158 and GSE191009 datasets were downloaded from the GEO database (https://www.ncbi.nlm.nih.gov/geo/).
