# Supplementary material for: ACLY Safeguards Copper Homeostasis to Sustain Brown Adipose Tissue Thermogenesis and Restrain Diet-Induced Obesity
Source: Research (Wash D C). 2026 May 7;9:1272. doi: 10.34133/research.1272 (PMC13150074; doi:10.34133/research.1272)
Supplement: Supplementary 1 — Figs. S1 to S5 Table S1 [file research.1272.f1.doc]

**Supplementary information**

**ACLY Safeguards Copper Homeostasis to Sustain Brown Adipose Tissue Thermogenesis and Restrain Diet-Induced Obesity**

Xin Liu, Lin Jia, Luwen Li, Jingnan Huang, Mengyun Hou, Zhijie Li, Dahong Yao, Yunfan Yang, Jigang Wang, Lingyun Dai

**This file includes:**

Figures. S1 to S5

Table. S1


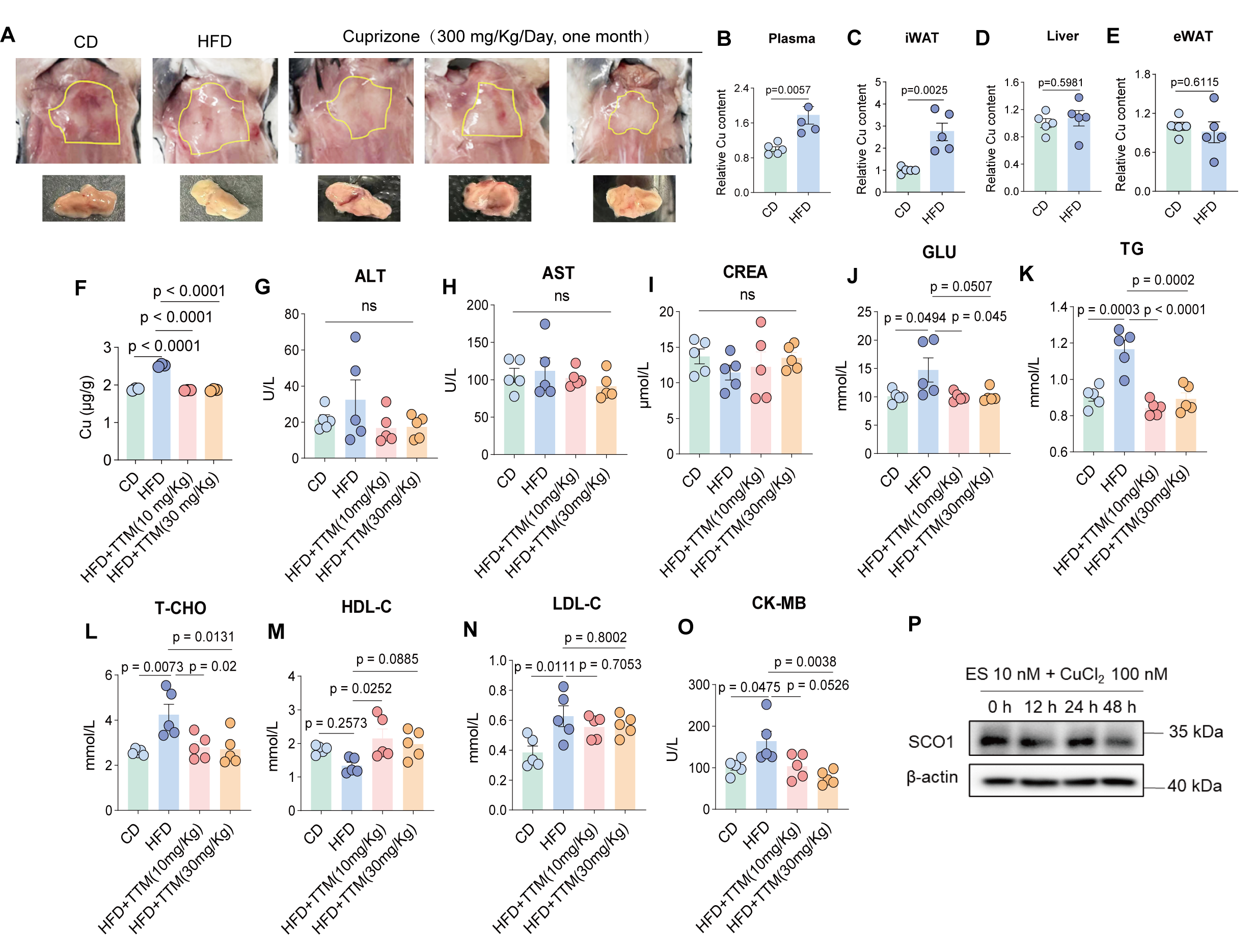


**Fig. S1.** **The beneficial effects of** **TTM on the systemic metabolism of DIO mice**. **(A)** Visualization of adipose tissue localized in the interscapular region and brown adipose tissue (BAT) of CD, DIO, and Cuprizone-treated mice. **(B-E)** Relative copper content in the liver, plasma, inguinal WAT (iWAT), and epididymal WAT (eWAT) of CD and DIO mice, n = 5. **(F)** The levels of copper content in the BAT of CD, DIO, and TTM-treated mice, respectively, which were determined using ICP-MS and normalized against tissue weight. **(G-O)** Serum levels of alanine aminotransferase (ALT), aspartate aminotransferase (AST), creatinine (CREA), fasting blood glucose (GLU), triglycerides (TG), total cholesterol (T-CHO), high-density lipoprotein cholesterol (HDL-C), low-density lipoprotein cholesterol (LDL-C), and creatine kinase MB isoenzyme (CK-MB) in CD, DIO group, and TTM-treated group mice, n = 5. **(P)** Analysis of SCO1 protein expression in primary brown adipocytes treated with 10 nM Elesclomol (ES) and 100 nM CuCl₂ for varying durations. Data are presented as the mean ± SEM of independent biological replicates; p < 0.05 represents a statistically significant effect, while ns represents no significant effect.


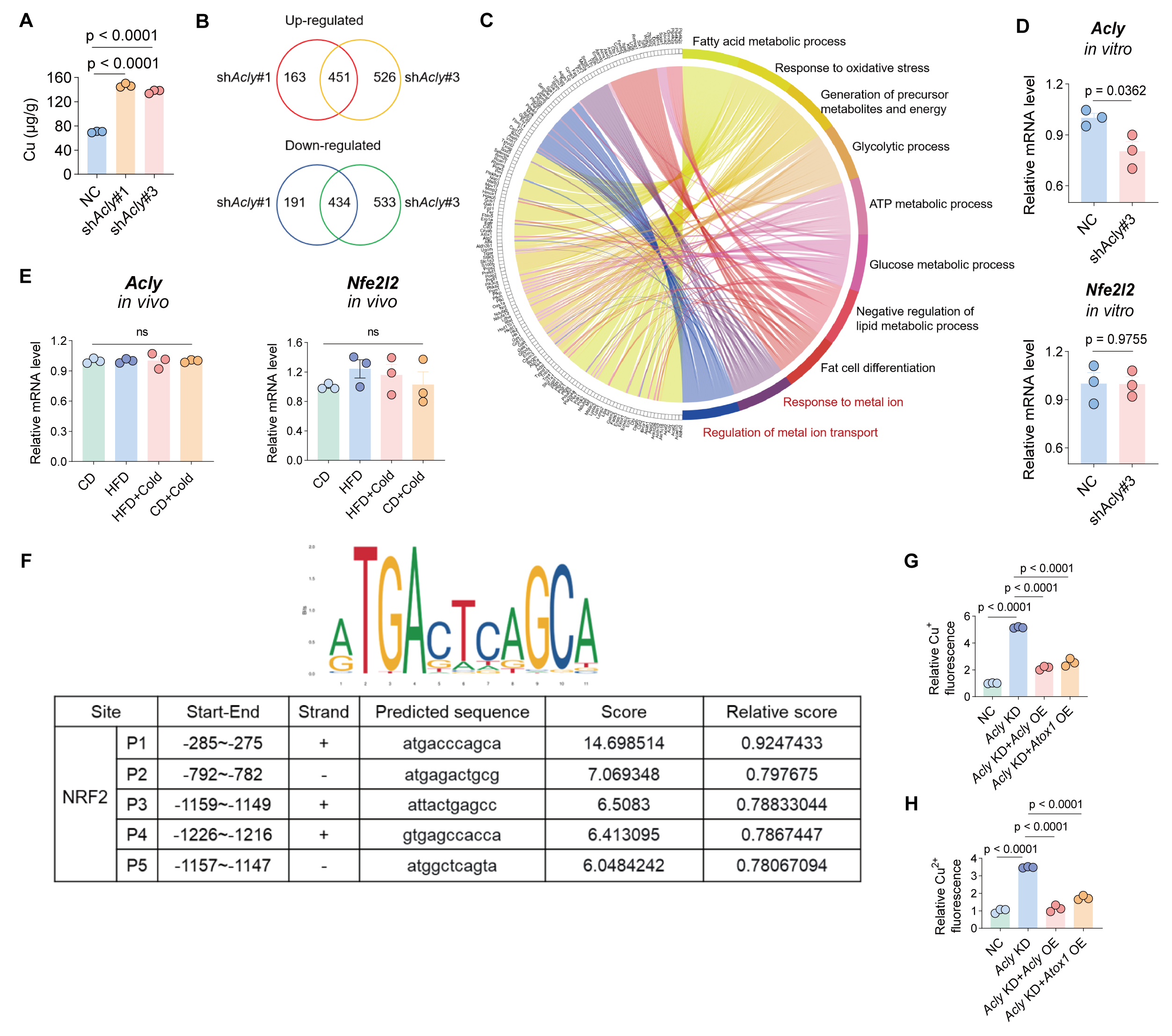


**Fig. S2.** The regulation of ACLY expression on NRF2 expression and ATOX1 transcription. **(A)** Using ICP-MS to determine copper content in *Acly*-knockdown C3H10T1/2 cells, normalized against protein content. **(B)** Venn diagram showing upregulated and downregulated differential proteins between two *shAcly*-knockdown cell lines and negative control (NC) cells. **(C)** Chord diagram of biological functional enrichment analysis for all DEPs in two *shAcly*-knockdown cell lines, with proteins on the left and pathways on the right. **(D)** Relative mRNA levels of *Acly* and *Nfe2l2* in *Acly*-knockdown C3H10T1/2 cells, n = 3. **(E)** Relative mRNA levels of *Acly* and *Nfe2l2* in the BAT of CD, DIO, cold-exposed DIO mice, and cold-exposed CD mice, respectively, n = 3. **(F)** The NRF2 (MA0150.3) binding motif on the *Atox1* promoter was predicted by the JASPAR database (<https://jaspar.elixir.no/>), with the relative confidence threshold set at 70%. **(G-H)** Statistical results of Cu²⁺ and Cu⁺ levels in cells from various groups, n = 3.


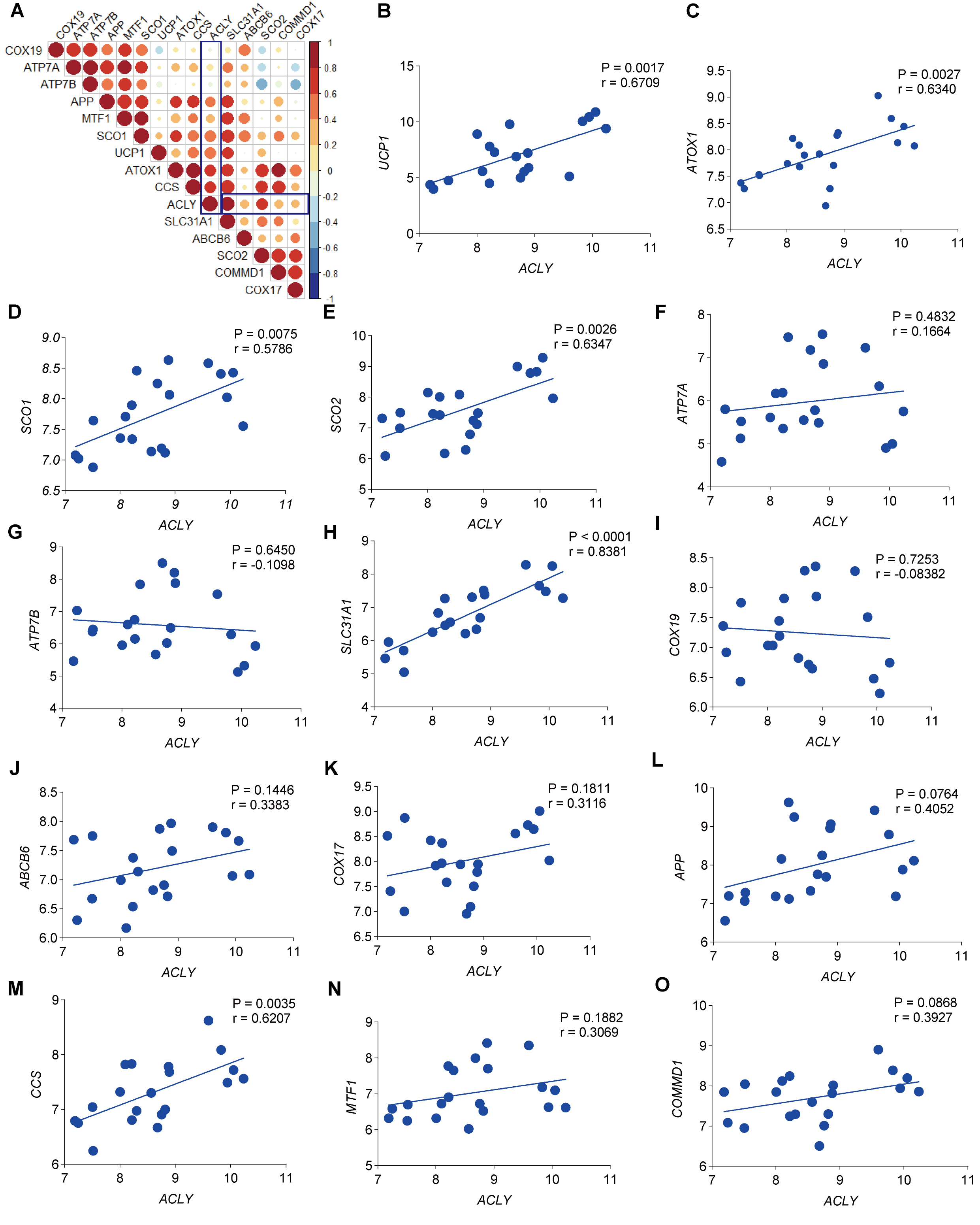


**Fig. S3. Correlation analysis of ACLY expression with the indicated genes in the BAT transcriptome of patients with metabolic disorders. (A)** Heatmap of correlation analysis between ACLY and UCP1, as well as copper-related genes (including ATOX1, SCO1, SCO2, ATP7A, ATP7B, SLC31A1, COX19, ABCB6, COX17, APP, CCS, MTF1, and COMMD1). **(B-O)** Pearson correlation scatter plots between ACLY and each of the aforementioned proteins.


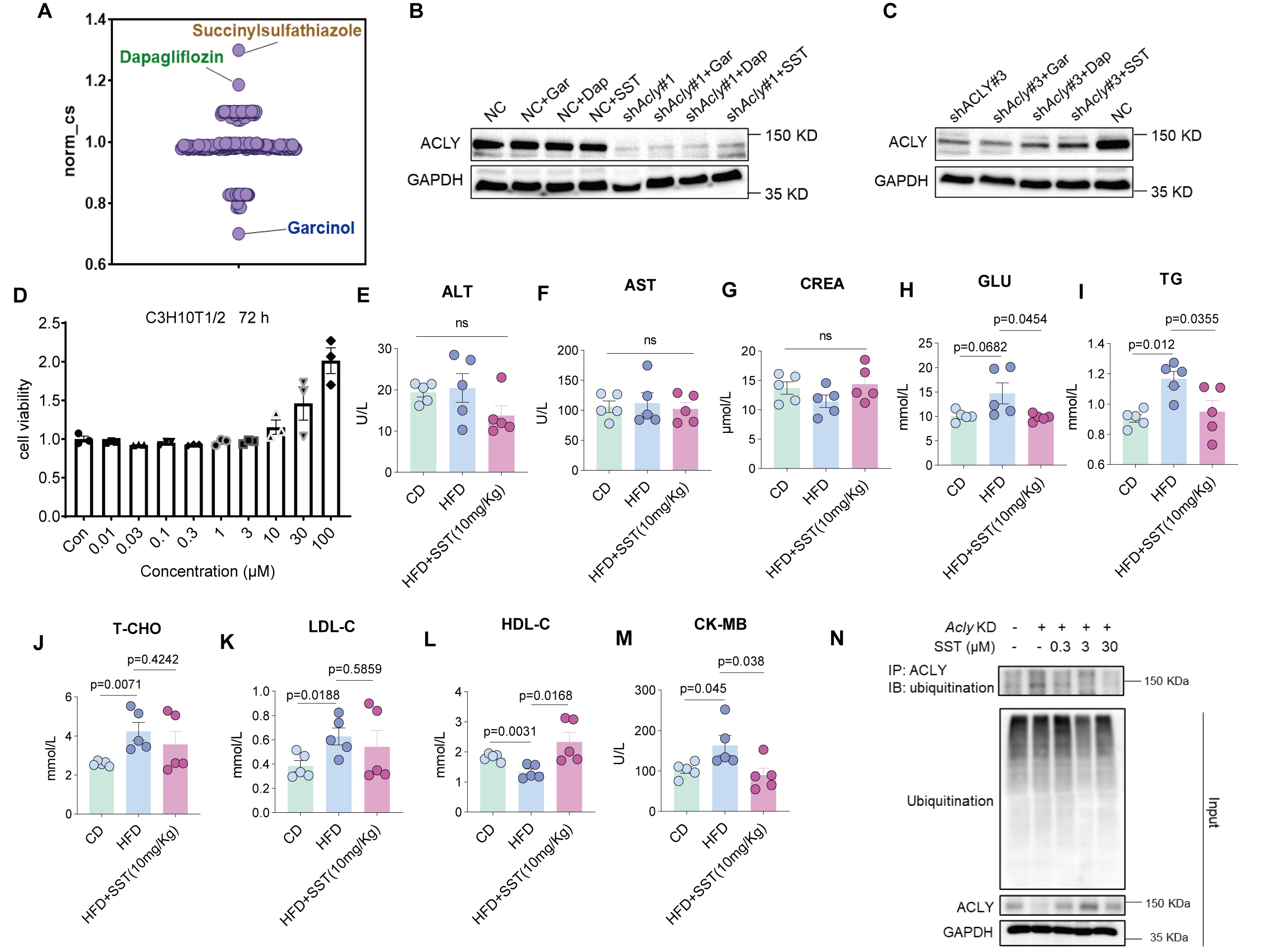


**Fig. S4.** **The beneficial effects of** **SST on the systemic metabolism of DIO mice.** **(A)** Small-molecule compounds potentially affecting ACLY expression, screened via the CMap database. **(B)** ACLY protein expression in normal control (NC) cells and *Acly*-knockdown cells treated with 10 μM of Succinylsulfathiazole (SST), Dapagliflozin (Dap), and Garcinol (Gar). **(C)** ACLY protein expression in another *Acly*-knockdown cell line treated with SST, Dap, and Gar (10 μM each). **(D)** Viability of C3H10T1/2 cells treated with varying concentrations of SST for 72 hours. **(E-M)** Serum levels of ALT, AST, CREA, blood glucose (GLU), triglycerides (TG), total cholesterol (T-CHO), low-density lipoprotein (LDL), high-density lipoprotein (HDL), and creatine kinase MB isoenzyme (CK-MB) in CD, DIO, and SST-treated DIO mice, n = 5. **(N)** Immunoprecipitation assay assessing the effect of varying concentrations of SST (0.3 μM, 3 μM, 30 μM) on the ubiquitination level of ACLY in *Acly*-knockdown C3H10T1/2 cells. Data are presented as the mean ± SEM of independent biological replicates; p < 0.05 represents a statistically significant effect, while ns represents no significant effect.


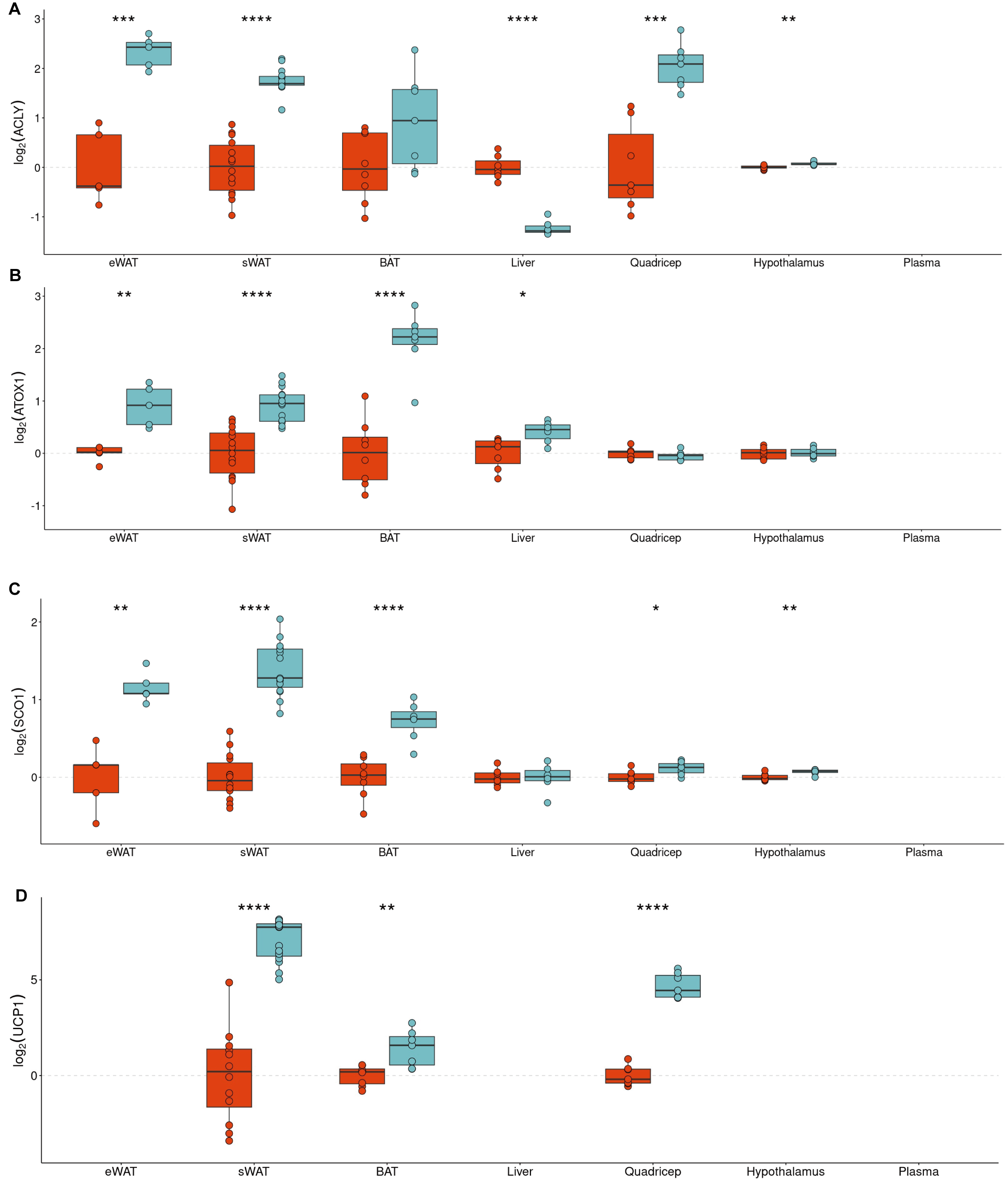


**Fig. S5.** **The changes in the expression levels of ACLY, ATOX1, SCO1, and UCP1 proteins in multiple tissues of mice under cold exposure stimulus**. Proteomics of the tissue of mice that accept cold adaptation. The relative expression changes of ACLY **(A)**, ATOX1 **(B)**, SCO1 **(C)**, and UCP1 **(D)** in epididymal white adipose tissue (eWAT), subcutaneous white adipose tissue (sWAT), BAT, Liver, Quadricep, Hypothalamus, and Plasma (retrieved from https://bigproteomics.shinyapps.io/ColdAdaptation/).

**Table S1**

| **REAGENT or RESOURCE** | **SOURCE** | | **IDENTIFIER** |
| --- | --- | --- | --- |
| **Antibodies** | | | |
| Rabbit polyclonal anti-UCP1, WB, dil:1/1,000 | PROTEINTECH | | Cat #23673-1-AP;  RRID: AB_2828003 |
| Mouse monoclonal anti-PGC-1α, WB, dil:1/1,000 | PROTEINTECH | | Cat #66369-1-Ig;  RRID: AB_2828002 |
| Rabbit polyclonal anti-ACLY, WB, dil:1/1,000 | PROTEINTECH | | Cat #15421-1-AP;  RRID: AB_2223741 |
| Rabbit polyclonal anti-SCO1, WB, dil:1/1,000 | PROTEINTECH | | Cat #12614-1-AP;  RRID: AB_2184641 |
| Rabbit polyclonal anti-SLC31A1, WB, dil:1/1,000 | PROTEINTECH | | Cat #27499-1-AP;  RRID: AB_2918124 |
| Mouse monoclonal anti-GAPDH, WB, dil:1/50,000 | PROTEINTECH | | Cat #60004-1-Ig;  RRID: AB_2107436 |
| Mouse monoclonal anti-Beta Actin, WB, dil:1/20,000 | PROTEINTECH | | Cat #66009-1-Ig;  RRID: AB_2687938 |
| Rabbit polyclonal anti- Histone H3, WB, dil:1/2,000 | PROTEINTECH | | Cat #17168-1-AP;  RRID: AB_2716755 |
| Rabbit monoclonal anti-ubiquitin, WB, dil:1/1,0000 | PROTEINTECH | | Cat #80992-1-RR;  RRID: AB_2923694 |
| Rabbit monoclonal anti-AMPKα, WB, dil:1/1,000 | Cell Signaling Technology | | Cat #5831T;  RRID: AB_2881899 |
| Rabbit monoclonal anti-Phospho-AMPKα (Thr172), WB, dil:1/1,000 | Cell Signaling Technology | | Cat #2535S;  RRID: AB_331250 |
| Mouse monoclonal anti-NRF2, WB, dil:1/1,000; IP, dil:1/200 | Santa cruz biotechnology | | Cat #sc-365949;  RRID: AB_2833793 |
| Mouse monoclonal anti-ATOX1, WB, dil:1/1,000; IF, dil:1/200 | Santa cruz biotechnology | | Cat #sc-100557;  RRID: AB_3671377 |
| Mouse monoclonal anti-ATP7A, WB, dil:1/1,000 | Santa cruz biotechnology | | Cat #sc-376467;  RRID: AB_2841712 |
| Rabbit polyclonal anti-ACLY, WB, dil:1/1,000 | HUABIO, China | | Cat #ET1609-37;  RRID: AB_2223741 |
| Mouse monoclonal anti-NRF2, WB, dil:1/1,000; IF, dil:1/1,000 | Servicebio, China | | Cat #GB113808;  RRID: AB_2833793 |
| Mouse monoclonal anti-Acetyllysine, WB, dil:1/1,000; IP, dil:1:200 | PTM Bio | | Cat #PTM-105RM;  RRID: AB_2841712 |
| Alexa Fluor™ 488 donkey anti-rabbit IgG (H+L), IF, dil:1/100 | Invitrogen | | Cat #A21206  RRID: AB_2556546 |
| Alexa Fluor™ 594 donkey anti-mouse IgG (H+L), IF, dil:1/100 | Invitrogen | | Cat #A21203  RRID: AB_2556543 |
| HRP-labeled Goat Anti-Mouse IgG(H+L), IF, dil, 1/500 | Servicebio, China | | Cat #GB23303  RRID: AB_2722565 |
| HRP-labeled Goat Anti-Rabbit IgG(H+L), IF, dil, 1/500 | Servicebio, China | | Cat #GB23301  RRID: AB_2839429 |
| **Chemicals, Peptides, and Recombinant Proteins** | | | |
| BioTracker Green Copper Live Cell Dye | Sigma-Aldrich | | Cat #SCT041 |
| Tetrathiomolybdate | Sigma-Aldrich | | Cat #323446-1G |
| CuCl_2_· 2H_2_O | Sigma-Aldrich | | Cat #10125-13-0 |
| XTHF60 diet | XIETONG SHENGWU, China | | Cat #XTM04-001 |
| Oil Red O | Servicebio, China | | Cat #G1015 |
| Succinylsulfathiazole | MedChemExpress | | Cat #HY-B0921 |
| Elesclomol | MedChemExpress | | Cat #HY-12040 |
| Rhodamine B hydrazide | MedChemExpress | | Cat #HY-123645 |
| DAPI | Servicebio, China | | Cat #G1012 |
| iF488-Tyramide | Servicebio, China | | Cat #G1231 |
| iF555-Tyramide | Servicebio, China | | Cat #G1233 |
| iF647-Tyramide | Servicebio, China | | Cat #G1232 |
| Gapdh | Sangon Biotech | | Cat #B661304-0001 |
| **Critical Commercial Assays** | | | |
| Copper (Cu^2+^) Colorimetric Assay Kit | Elabscience, China | | Cat #E-BC-K300-M |
| Cell Copper (Cu^2+^) Colorimetric Assay Kit | Elabscience, China | | Cat #E-BC-K775-M |
| Cellular Cuprous Fluorometric Assay Kit | Elabscience, China | | Cat #E-BC-F102 |
| Amplex Red Cholesterol and Cholesteryl Ester Assay Kit | Beyotime, China | | Cat #S0211S |
| SYBR-green PCR mix | Vazyme, China | | Cat #Q511-02 |
| BCA assay kit | ThermoFisher Scientific | | Cat #A55864 |
| BeyoChIP™ Enzymatic ChIP Assay Kit | Beyotime | | Cat #P2083S |
| **Experimental Models: Organisms/Strains** | | | |
| Mouse C57BL/6J | GemPharmatech | | Cat #N000013  RRID：IMSR_JAX:000664 |
| **Primers used for qPCR** | **Forward primer** | | **Reverse primer** |
| *Acly* | TTCGTCAAACAGCACTTCC | | ATTTGGCTTCTTGGAGGTG |
| *Nfe2l2* | CACATCCAGTCAGAAACCAGTGG | | GGAATGTCTGCGCCAAAAGCTG |
| *Atox1* | ATGCCGAAGCACGAGTTCTC | | ATGCAGACCTTCTTGTTGGGC |
| **Sequences of shRNA** | | | |
| **sh*Acly*#1** | 5’-CCGGGCTGAATACCGAGGACATTAACTCGAGTTAATGTCCTCGGTATTCAGCTTTTTG-3’ | | |
| **sh*Acly*#2** | 5’-CCGGGCATGAGGTCACCATCTTTGTCTCGAGACAAAGATGGTGACCTCATGCTTTTTG-3’ | | |
| **sh*Acly*#3** | 5’-CCGGGGAAGCTGATGAATATGTTGACTCGAGTCAACATATTCATCAGCTTCCTTTTTG-3’ | | |
| **Primers used for ChIP-qPCR** | **Forward primer** | **Reverse primer** | |
| **P1** | CCTTGGCCTTGCATTCCCTA | TGCTTACCGTGCCTTTCGAT | |
| **P2** | TAATTGGGTGGGAAGGACGC | GGTGGTCATGCCTTCCTTCA | |
| **P3/P5** | GCTGGGATTTGAACTCGGGA | TGGAGGCTGAGAGGAGATCC | |
| **P4** | CCTCTGGAGTGTAGGGGGAA | CCGACTGCTCTTCCAAAGGT | |
